# Monitoring Potentially Toxic Element Pollution in Three Wheat-Grown Areas with a Long History of Industrial Activity and Assessment of Their Effect on Human Health in Central Greece

**DOI:** 10.3390/toxics9110293

**Published:** 2021-11-04

**Authors:** Georgios Thalassinos, Vasileios Antoniadis

**Affiliations:** Department of Agriculture Crop Production and Rural Environment, School of Agricultural Sciences, University of Thessaly, Fytokou Street, 384 46 Volos, Greece; thalassinosgeorgios@hotmail.gr

**Keywords:** PTE exposure, hazard index, metal accumulation, metal bioavailability, wheat consumption

## Abstract

Agricultural lands, especially those where wheat is cultivated, in the vicinity of intense anthropogenic activities may be laden with potentially toxic elements (PTEs), resulting in increased risk for human health. In this study we monitored three regions located in central Greece, currently cultivated with wheat: Domokos and Eretria, two areas with abandoned chromium mines, but never studied before, and the industrial area of Volos, near a major steel factory. All soils were alkaline with medium CaCO_3_ content. As expected, Cr was extremely high in the first two areas (705.2 in Eretria and 777.5 mg kg^−1^ in Domokos); Ni was also found elevated (1227 in Eretria, 1315 in Domokos and 257.6 mg kg^−1^ in the steel factory), while other harmful metals (Cd, Cu, Pb and Zn) were rather low. As a result, pollution load index, a cumulative index showing the contamination level of an area, was higher than 1.0 in all three areas (Eretria = 2.20, Domokos = 2.28, and steel factory = 1.61), indicating high contamination and anthropogenic inputs. As for the wheat parts (shoots and grains), they were found to have no elevated concentrations of any of the measured metals in all three study areas, probably due to the alkaline soil pH that decelerates metal mobility. This was also confirmed by the very low soil-to-plant transfer coefficient values for all metals. In assessing the possible risk concerning human health, we found that the soil-to-human pathway would induce no significant risk (exhibited by hazard index of less than 1.0), while the risk from grain-to-human resulted in considerable risk for human health in the steel factory of Volos (where HI > 1.0). Our findings suggest that rural areas never studied before with a history in some offensive anthropogenic activity can prove to be a contamination hotspot; we regard this study as a pivotal for similarly never-visited-before areas casually cultivated with wheat (or other important crops for human nutrition). We further recognize the need for a more in-depth study that would acknowledge the geochemical speciation of the studied metals and also monitor other important crops and their possible uptake of PTEs.

## 1. Introduction

Potentially toxic elements (PTEs) in soil may be of lithogenic or anthropogenic origin. Surface soils are considered to be the main receptors of PTEs emitted to the environment from various sources. Natural procedures such as mineral weathering, volcanic eruptions and erosion may increase soil PTE concentrations [1]. On a large scale the lithogenic derivation of PTEs is the most important factor affecting PTE concentrations in topsoil. On the other hand, anthropogenic PTE inputs are related mainly to industrial activities, such as mining, electroplating, smelting, wastewater discharge and aerial deposition of industrial fumes [2]. Other xenobiotics (such as plant protection products, fertilizers or pharmaceuticals), are eventually also deposited into soil as a result of human activities and are likely to induce problems and risks [3,4]. Within the past decades, agricultural activities have also enhanced PTE emissions; such activities involve excessive use of chemical fertilizers, pesticides, and the use of organic wastes of high macronutrient content, applied to soil so that the agricultural products may meet the ever increasing food demands. Such activities, whether industrial or agricultural, have resulted in elevated PTE concentrations in agricultural soils [5]. High concentrations of PTEs in agricultural soils may result in toxic effects to humans via the consumption of cultivated crops, pollution of the ground water or even direct soil particles ingestion [6]. Indeed, a number of metallic PTEs, including Cd, Co, Cr, Cu, Fe, Mn, Ni, Pb and Zn, may exert adverse health effects on human health, if their concentration exceeds some critical values. In agricultural areas in particular, the induced risks of high metal contents may lead to an imbalance of the ecosystem services, an unwanted outcome, given the high anthropogenic inputs in energy and resources in such environments. Extensive analysis of experimental results has led to the establishment of critical concentrations for a series of PTEs concerning environmental and human health impact, and their environmental fate has been extensively investigated due to the non-biodegradable nature of PTEs and the potential human health risk involved (including the pathways of consumption of PTE-contaminated food, soil particle ingestion, dermal absorption and soil particle inhalation) [7,8].

Plants cultivated in soils polluted with PTEs tend to develop mechanisms to limit their uptake and prevent the translocation of PTEs from root to aerial tissues [9]. However, a major route of human exposure relates to the consumption of plant tissues with high concentrations of PTEs. Plant tissue PTE concentrations relate to numerous soil properties that regulate PTE phytoavailability. In particular, soil parameters such as soil activity (pH), soil organic matter, soil texture, redox conditions and the concentration of competing cations significantly affect reactions and processes such as PTE adsorption, precipitation and the formation of ligand complexes that significantly affect PTE availability [10]. 

In recent years there have been some works monitoring soil PTE levels in central Greece [11]; however, there are areas that have never been studied, although they are suspected as being of high contamination load. These areas are being routinely cultivated with important food crops, such as wheat, for the production of flour for bread and pasta making. Two such areas are in Domokos (Prefecture of Fthiotida) and in Eretria (Prefecture of Larissa, Thessaly): they both have a decades-long history of now-ceased mining exploitation due to the fact that there were economically significant refractory type chromite ore deposits [12]. Minerals associated with chromite crystals such as (Mg,Fe^3+^)(Cr,Al,Fe^3+^)_2_O_4_ vary widely, including olivine, pyroxene, serpentine, magnetite ferrochromite, chlorite, talc and magnesite. Interstitial Ni and Cu base-metal sulfide alloys may also be present (e.g., (Ni,Fe^2+^)_7_S_6_, (Ni,Fe,Co,Cu)Ni_2_S_4_, and (Ni,Fe)_9_S_8_) [13]. Chromite samples from New Zealand had Cr_2_O_3_ content ranging from 25.5 to 57.4%, while Ni ranged from 1000 to 1700 mg kg^−1^ and Pb was as low as 0.531 mg kg^−1^ [14]. In northern Greece where metamorphosed laterites occur, Fe_2_O_3_ concentrations were found to range from 44.4 to 67.80, while Cr ranged from 7400 to 91,000, Ni 1000–16,000, Co 180–1200 and Zn 60–850 mg kg^−1^ [15,16]. In Eretria (Mount Othris, Greece), Cr was very high (8950–11,270), while Ni was as low as 10–50 mg kg^−1^ [11]. Furthermore, chromium rich parent matrix has been referred to release dust particles that contain high levels of Fe and Mn, resulting in heavy metal pollution dispersal [17].

In recent years, there has been a major concern that past but intense industrial activity may result in enhanced soil PTE concentrations and that, in turn, cultivated wheat may also be affected. However, to our knowledge, these important wheat-producing areas have never been studied before; there indeed is a great necessity to assess the PTE exposure pathways to humans and the related PTE-induced health risk assessment. There is also a third area in central Greece which is of major interest: the industrial area of Volos (Prefecture of Magnesia, Thessaly), especially the agricultural land adjacent to a major steel factory. Such industries are known to induce heavy metal pollution. In an industrial city, Anshan, Liaoning, Northern China, a study showed that areas around steel factories could be characterized as pollution hotspots and PCA indicated that emissions led to an increase in soil Cu, Zn, Pb and Cd [18]. The same trend was noticed in a metal pollution investigation around a steel factory in Middletown Ohio for Ni, Cr, and Zn [19]. In yet another monitoring assay around steel factories, Pb levels in soil were found to be similarly high [18], and the contamination factor (CF) equaled 4.7 in street dust [19]. The third site of interest in our work has recently been monitored [20] and health impact through soil ingestion and the consumption of another important crop of that area, maize, was estimated [21]. However, in our work we extended the monitoring of this area by studying the PTE effect in the particular spots of that site where wheat is cultivated. To the best of our knowledge, this is the first work that attempts to monitor three areas currently cultivated with wheat, all with a history in offensive industrial activities and thus suspected with elevated soil PTE contents; the risk of major PTEs entering the human food chain via wheat grain consumption and the assessment of the associated human health risk is also novel, especially in these particular study areas, never visited before, although long suspected as contamination hotspots. The aims of this study were the (a) determination of the level of contamination by PTEs in soil, pseudo-total and available, and in wheat in three important wheat-cultivated areas of central Greece with a known history of offensive industrial activity, (b) use of various contamination indices for assessing the level of PTE contamination to soil and plant, and (c) assessment of human health risk related to PTEs through wheat consumption and soil ingestion from the studied areas. We studied 9 PTEs which are considered to be very important in similar studies, i.e., Cd, Co, Cr, Cu, Fe, Mn, Ni, Pb, and Zn. The specific goals set in this work were to monitor soil PTEs in comparison to their background concentrations, to test whether a major crop in these areas, i.e., wheat, is being affected, and to assess the induced health risk.

## 2. Materials and Methods

### 2.1. Site Description 

**Figure 1:**
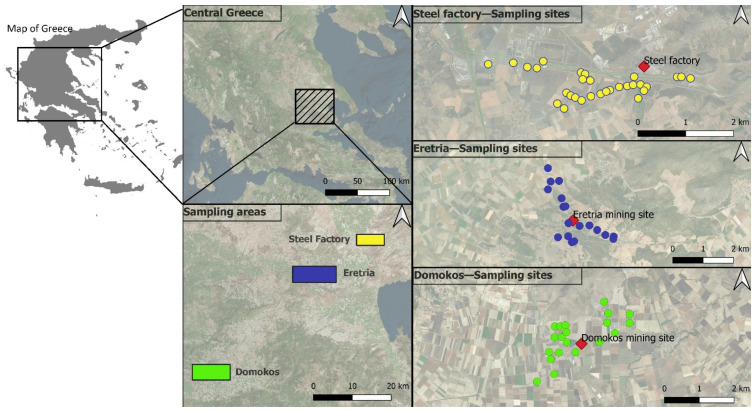
Map of the locations of the three study areas and the sampling points in central Greece.

Topsoil and aerial plant samples were collected from a total of 68 locations in central Greece (Eretria, Domokos, and the industrial area of Volos immediately adjacent to the steel factory). The three areas share similar climatic conditions, which is typical Mediterranean, with hot dry summers and cold and wet winters. Domokos has a 30-year average of 517 mm of precipitation and 18.7 °C of mean temperature. Eretria has 339 mm of precipitation and 22.8 °C of mean temperature. Volos has 587 mm and 21.7 °C of mean temperature. The area lacks a soil inventory with recorded background metal contents. Eretria soils were described as Leptosols and Calcisols (Table S1; Sampling positions in Eretria and soil physicochemical characteristics.), those in Domokos as Cambisols and Vertisols (Table S2), and those in the steel factory of Volos as Cambisols (Table S3). No detailed information concerning the soil profiles of the sampling points were obtained. The lithology of the three study areas include mafic and ultramafic rocks, as well as metamorphic rocks, mainly calcitic and dolomitic marbles, phyllites, and psammites. In Tables S1–S3, the exact geographical positioning of the samples is indicated, and the map of the sampling points is shown in Figure 1.

### 2.2. Soil and Wheat Sampling and Processing

Samples were collected just before the harvest of wheat crops, in May 2019. We sampled plants were at a BBCH stage 89, referred to as “fully ripe.” For each sampling site, a soil sample (0–5 cm) of ca. 1 kg was obtained after pebbles and vegetation were removed and plant samples (wheat shoots and wheat grain samples) were obtained from exactly the same spots where soils were obtained. This depth of soil sampling was preferred based on (a) the fact that wheat roots are mostly distributed near the surface, thus sampling at deeper layers would lead to a “dilution” of metal contents and thus an underestimation of their effect, and (b) any new/recent metal depositions are expected to be anthropogenic, and thus likely to occur from surface down (contrary to lithogenic inputs, which are likely to occur from deeper layers up). More specifically, from Eretria we collected samples from 18 locations. From Domokos, samples were collected from 20 locations and, from the industrial area of Volos, samples were collected from 30 sites (resulting in a total of 68 sampling points; Figure 1; coordinates of the sampling sites, as well as characterization analyses are presented in Table 1 and Tables S1–S3). The fields, according to personal communication with local farmers, have been cultivated with wheat for several decades now, and typical yearly fertilizer input is equivalent to 100 units of N, 20–30 units of P_2_O_5_ and 20–30 units of K_2_O (units are kg ha^−1^).

From each of the 68 sampling points, we collected 10 different plants, cut at 10 cm above soil surface. The plants were at full maturity, only days before harvest. Immediately after sampling, the plants were taken to the laboratory and separated into grains and the rest of the plant (thereafter referred to as “shoots”). As for soil, four soil subsamples were taken from the edges of a square block (1 m × 1 m) placed around the plant sampling site and were thoroughly mixed to form one composite soil sample. Soil samples were left to air dry, then placed in a forced draught oven at 100 °C until no further weight loss was noticed and were sieved through a 2 mm sieve. Plant samples were acid-washed with 2% HNO_3_, rinsed with distilled water, left to air dry, placed in a force draught oven at 70 °C until no weight loss was noticed and milled to fine powder with a nonmetallic grinding mill.

### 2.3. Soil and Plant Analyses

For the extraction of the pseudototal element concentrations in soil samples, aqua regia (1 g of soil digested into 20 mL of conc. HCl/HNO_3_ at 3/1 for 3 h) was used and digestion was performed using a Velp DK 20 digestion unit. The phytoavailable PTE soil concentrations were assessed with extraction with DTPA-TEA-CaCl_2_ pH 7.3 (1:2 soil-to-DTPA shaken for 2 h). To estimate the concentration of different elements on plant tissues, 1.00 g of the finely ground plant sample was dry-ashed at 500 °C for 5 h and was extracted using 20 mL of 20% HCl into 50-mL volumetric flasks [22]. Soil and plant tissue PTE concentrations were assessed using a flame atomic absorption photometer (Perkin Elmer 3300).

### 2.4. Indices

#### 2.4.1. Contamination Indices

The following contamination indices were estimated [23,24,25]:

1. Transfer coefficient

(TC) = C_P_/C_S_

where C_P_ = PTE concentration in plants and C_S_ = aqua regia-digested concentrations of PTE in soil;

2. Contamination factor

(CF) = (concentration of a PTE in the soil sample)/(global average background concentration of the same PTE)

The background values we used were those reported as “world average values” by Kabata-Pendias [26], and were as follows: Cd (0.41), Co (11.3), Cr (59.5), Cu (38.9), Fe (non-existent in Kabata-Pendias [26]), but taken equal to 20,000), Mn (480), Ni (29), Pb (27), and Zn (70) (values in mg kg^−1^). Using the CF, soil samples can be classified as follows: soil is of low contamination if CF < 1; of moderate contamination if 1 < CF ≤ 3; of considerable contamination if 3 < CF ≤ 6; and of very high contamination if CF ≥ 6;

3. Pollution load index

(PLI) = (CF_1_ × CF_2_ × CF_3_ … × CF*_n_*)^1/*n*^

The pollution load index is used to calculate the degree of soil pollution taking into consideration the CF values of *n* PTEs in a monitored area (in our work, *n* = the number of studied PTEs = 9). Soil samples can be categorized as being below the baseline of pollution if PLI < 1, while PLI > 1 exhibits a soil polluted beyond the baseline level.

#### 2.4.2. Health Risk Assessment Indices (Noncarcinogenic)

To assess health risk related to soil particle ingestion (i.e., via the soil-to-human pathway), the hazard quotient (HQ) was calculated as the ratio of direct intake of soil via ingestion (D_s-ing_) to the reference dose of oral human intake (RfD_0_) (HQ_ing_ = D_s-ing_/RfD_0_). 

D_s-ing_ is given by the following equation:

D_s-ing_ = C_s_ × ((IngR × EF × ED)/(BW × AT)) × 10^−6^

where IngR = ingested soil, taken equal to 100 mg of soil day^−1^ (for adults) and 200 mg of soil day^−1^ (children); EF = exposure frequency, equal to 350 days per year; ED = exposure duration, equal to 30 years (adults) and 6 years (children), BW = body weight, equal to 70 kg (adults) and 15 kg (children), AT = averaging time = EDx365 = 10,950 days (adults) and 2190 days (children); the factor of 10^−6^ is used for unit conversion. Reference dose for human oral intake (RfD_0_) were used as follows: 1.0 (Cd), 0.3 (Co), 1500 Cr, 40 (Cu), 700 (Fe), 140 (Mn), 20 (Ni), 3.5 (Pb) and 300 μg of PTE day^−1^ kg^−1^ BW (Zn). 

HQ relative to food consumption was calculated as the ratio of human PTE intake via food consumption (D_F_) to the maximum tolerable daily intake (TDI) (HQ_F_ = D_F_/TDI). Human consumption due to food intake (D_F_) was calculated as follows:

D_F_ = C_F_ × ((MIDVC × EF × ED)/(BW × AT)) × 10^−6^

where MIDVC = mean individual daily food consumption (taken equal to 0.3836 kg wheat per capita, as per USDA [27]); EF = exposure frequency (350 day yr^−1^); ED = exposure duration (ED = 30 years). TDI values were taken equal to 1.0 (Cd), 1.4 (Co), 300 (Cr), 140 (Cu), 700 (Fe), 140 (Mn), 2.8 (Ni), 3.5 (Pb) and 500 μg d^−1^ kg^−1^ (Zn).

Noncarcinogenic risk was then assessed using the hazard index (HI), which measures the cumulative effect of the studied PTE hazard quotients (HQ), as follows:

HI = Σ(HQ)

HI > 1 indicates significant health risk for humans, while HI < 1 indicates that human exposure is within acceptable levels [28].

### 2.5. Statistical Analysis and Data Quality Control

Figures were created using Microsoft Excel 2019 and IBM SPSS Statistics 25. Analysis of variance was calculated with IBM SPSS Statistics 25 at the level of significance of *p* < 0.05. Data quality control was addressed with the use of in-house reference soil and plant materials with each batch of extraction and analysis. When values fell outside the target of plus/minus 10% of the predetermined values, analyses were repeated. Blank samples were assessed for possible laboratory glassware contamination, and any estimated values were subtracted from each extraction batch. Each soil and plant sample was analyzed thrice, and values fell within RSD < 15%. Principal component analysis was performed: Correlation analysis of the studied variables (nine variables of total element concentration and 1 variable measuring the distance from the possible pollution source) resulted in a 10 × 10 correlation matrix for each studied area, rendering this technique impractical to summarize the effects of the variables [29]. PCA reduces the number of variables that describe the variance and correlations between the measured parameters into a minimal number of linearly independent variables, named principal components (PCs). Factor loading of the measured parameters on PCs can be classified as strong when >0.70, moderate for loadings ranging from 0.70–0.50 and weak for values < 0.50 [30]. We used SPSS (version 25) for PCA with Varimax rotation and figures illustrating the results were made using Excel 2020. PCA was conducted in order to identify the potential sources of heavy metals. 

## 3. Results 

### 3.1. Soil Physicochemical Characteristics 

Soils from Eretria and Domokos were slightly alkaline (with average pH of 7.19 and 7.34, respectively), of relatively low CaCO_3_ and soil organic carbon content, of loamy texture and had amorphous oxide concentration of average 57.11 and 78.36 mmol kg^−1^, respectively (Table 1 and Tables S1–S3). Soil samples from the vicinity of the steel factory (industrial area of Volos), were of higher pH (average pH = 8.1) and CaCO_3_ (average value 5.15%), while organic carbon content was still low (average value 1.25%); soils were mainly of clay loam texture and the average amorphous oxide concentration was 74.19 mmol kg^−1^. 

### 3.2. Total Contents of Trace Elements in Soil

**Figure 2:**
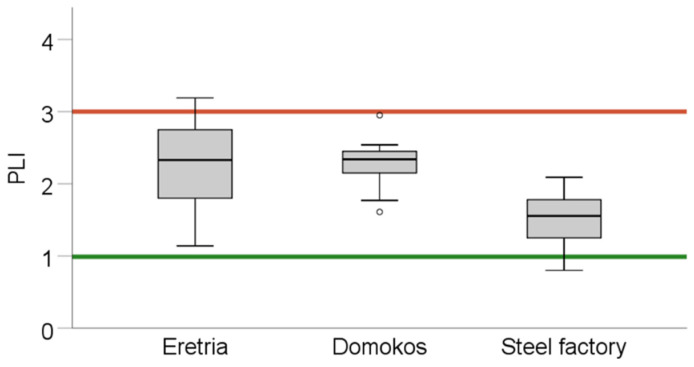
Pollution load index of the soils obtained from Eretria, Domokos and the industrial area of Volos (near the steel factory). Circles and asterisks denote outlier values.

The total concentrations of the nine studied elements (Cd, Co, Cr, Cu, Fe, Mn, Ni, Pb, and Zn) were measured in the soil samples, obtained from the three areas: Eretria, Domokos and steel factory (industrial area of Volos; Table 2 and Table S4). The studied metals varied widely within each site showing a considerable spatial distribution; to this the landscape of the areas did not seem to have played any role, due to the fact that the areas were rather flat with no significant relief. Total element content analysis in Eretria indicated that some PTEs were found to have higher than background values: Cd (average value = 0.51 vs. background value = 0.41), Co (68.38 vs. 11.3), Cr (705.2 vs. 59.5), Mn (1004 vs. 480) and Ni (1227.5 vs. 29, all units in mg kg^−1^); some of these elements, Co, Cr and Ni, they surpassed by far the maximum allowable concentrations (Table 2). Concerning Domokos, those with average values higher than the background concentrations were: Cd (average value of 0.45), Co (84.05), Cr (777.5), and Ni (1315; units in mg kg^−1^); similar to the case of Eretria, Co, Cr and Ni were the elements with extremely high contents, and also exceeded the maximum allowable concentrations. As for the pseudo-total PTEs of the Volos industrial area (steel factory), Co (26.4), Cr (359.4), Ni (257.6), and Zn (86.1; units in mg kg^−1^) exceeded the respective background values, while Cr and Ni were found to be very high. However, it should be noted that Zn and Cu were found in relatively low concentrations and that Pb maximum concentrations were lower than the global average background values. Other PTE-related parameters are also presented in the Supplementary Material (Tables S5–S9 and Figure S1). The cumulative effect of all studied PTEs, as assessed from the PLI, in Domokos, Eretria and the steel factory was 2.20, 2.28 and 1.61, respectively, higher than unity in all cases (Figure 2). 

### 3.3. Principal Component Analysis

**Figure 3:**
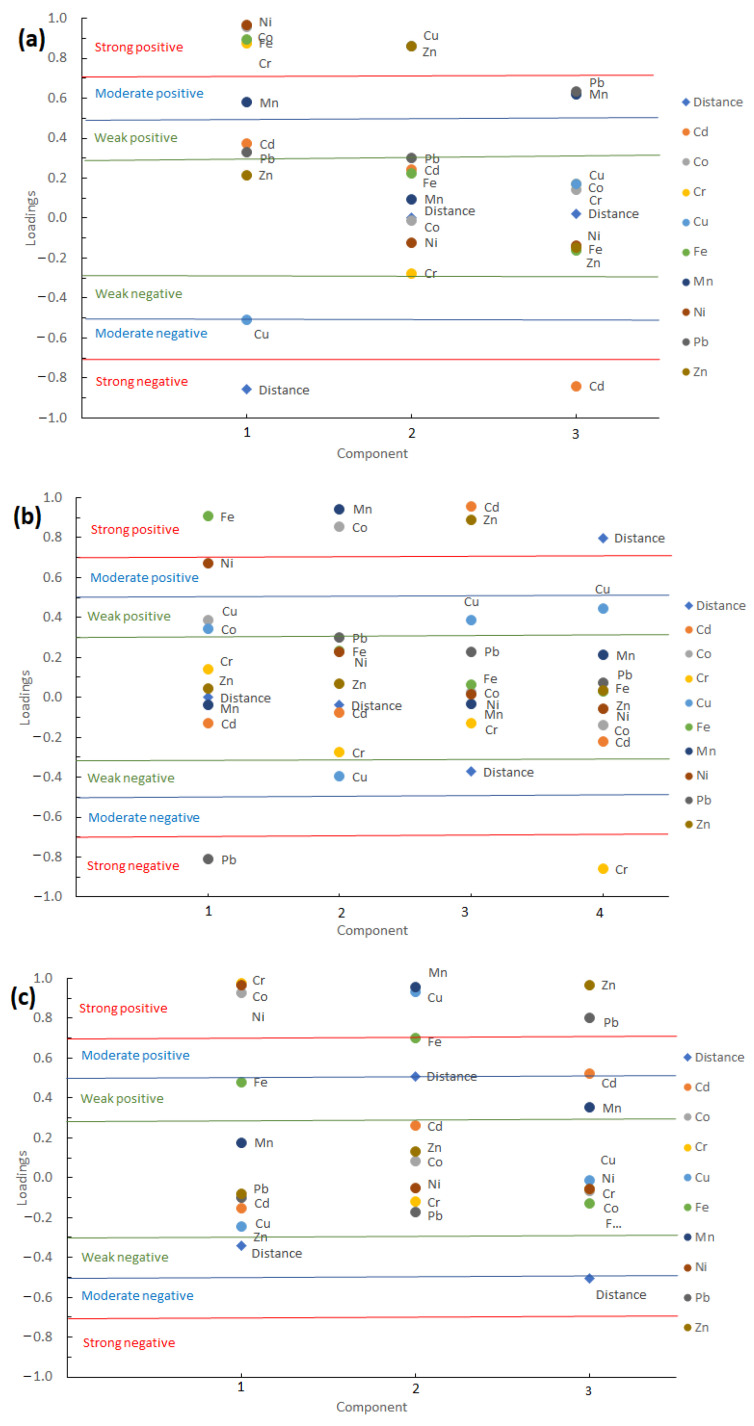
Loading plot for PCs extracted for (**a**) Eretria, (**b**) Domokos and (**c**) steel factory.

For the Eretria dataset, three principal components were extracted using the Eigenvalue—one criterion, explaining 84.273% of the total variance (51.21% PC1, 17.40% PC2 and 15.7% PC3) (Tables S10–S15; Figures S2–S4). Positive loading on PC1 was noticed for Ni, Co, Fe and Cr, while strong negative loading for the variable “distance” of each sampling site from the suspected pollution source. Cu and Zn were positively loaded on PC2 (Figure 3). For PC3 there is a moderate positive loading of Pb and a strong negative loading for Cd. For the Domokos dataset, four principal components were extracted, explaining 79.712% of the total variance (27.03% PC1, 21.74% PC2, 16.15% PC3, and 14.79% for PC4; Figure S3). For PC1, Fe and Ni were positively loaded and Pb had strong negative loading. Relative to PC2, Mn and Co were highly associated, and for PC3, Cd and Zn were positively associated. For the dataset of the Volos steel factory, three principal components were extracted, explaining 82.1% of the total variance (43.7% PC1, 23.8% PC2 and 14.6% PC3). We noticed that Cr, Co and Ni were positively associated to PC1 and Mn and Cu positively associated to PC2. For PC3, strong positive associations were noticed for Pb, Zn and moderate positive association for Cd, while weak-to-moderate negative loading was noticed for “distance” from the steel factory.

### 3.4. Wheat Tissue and Grain Potentially Toxic Element Concentrations and Soil-to-Plant Transfer

**Figure 4:**
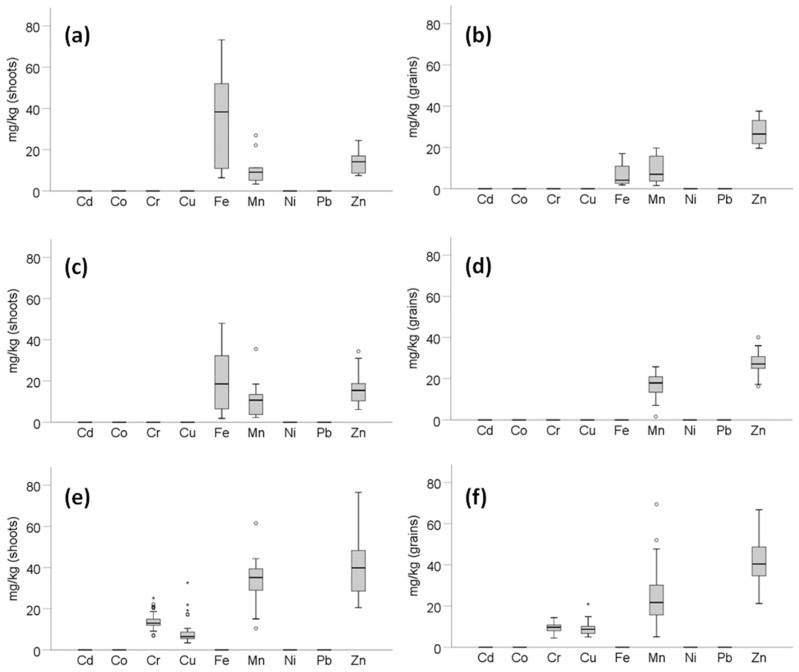
PTE concentrations in mg kg^−1^ dry mater of shoots and grains in Eretria (**a**,**b**), Domokos (**c**,**d**) and the steel factory (**e**,**f**) wheat samples. Circles and asterisks denote outlier values.

In Eretria and Domokos, Cd, Co, Cr, Cu, Ni and Pb concentrations were below detection limit (Figure 4; detailed data in Tables S16–S17). In the steel factory, likewise, Cd, Co, Fe, Ni, and Pb were below detection limits. Concerning Eretria, Mn in shoots and grains did not differ, while Fe in shoots was significantly higher than those in grains (*p* = 0.008); contrary to that, Zn in shoots was significantly lower compared to those in grains (*p* = 0.002). In Domokos, Mn and Zn were lower in shoots than in grains (*p* < 0.001). In the steel factory of Volos, Cr in grains was significantly lower than in shoots (*p* < 0.001), while Cu, Mn and Zn did not differ within the plant parts. Also available soil PTEs are shown in Table S18.

In our work, TC values (Table 3) are presented “per mille” (multiplied by a factor of 10^3^) for clarity of the reported numbers. Shoot Zn TC in the industrial area of Volos were the highest among the studied areas, with significant differences from that in Domokos and Eretria (*p* = 0.003). Likewise, Mn TC in wheat shoots were significantly lower in Domokos and Eretria, compared to the steel factory (*p* < 0.001). Overall, TC values in the steel factory were higher compared to those from Eretria and Domokos (Table 3). Comparing the TC values between shoots and grains, statistically significant differences were observed in Domokos for Mn and Zn (which were higher in grains) relative to the other areas; in the industrial area of Volos, Cr TC was significantly lower in grains than in shoots.

### 3.5. Hazard Index (HI) 

#### 3.5.1. Hazard Index for Soil Particle Ingestion

**Figure 5:**
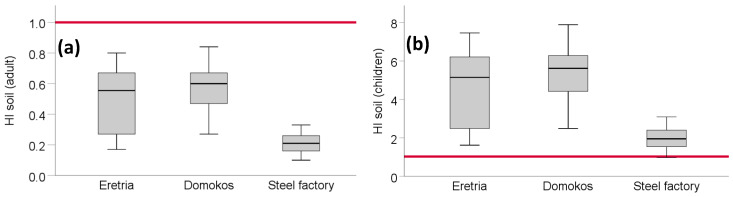
Hazard index (of the soil ingestion pathway) for adults (**a**) and children (**b**).

The most important contribution in HI was by Co, Fe, and Ni, which had significantly higher HQ values than the rest of the PTEs (*p* < 0.001), while for Mn, Pb, Cd, Cr, Cu and Zn minimal values were observed (Figure S5; Table S19–S21). The mean value of the total health risk (HI) for soil ingestion of adults was lower than unity, indicating that adult exposure to soil particle ingestion was unlikely to pose health threat to adults (Figure 5; Table S21). Contrary to that, the values for children were higher than unity for all three studied areas.

#### 3.5.2. Hazard Index for Wheat Grain Consumption

**Figure 6:**
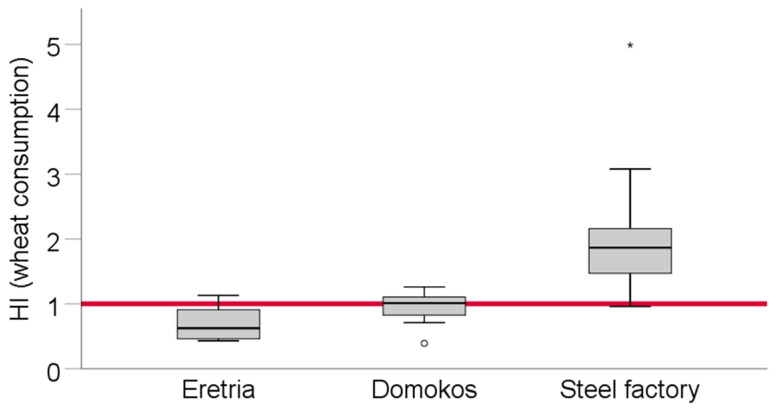
Hazard index for wheat grain product consumption. Circles and asterisks denote outlier values.

The health risk assessment via the food consumption pathway concerns by definition only adults, not children, due to the length of the potential exposure. In Eretria and Domokos the HI did not differ (*p* = 0.288), while that in the steel factory was significantly higher (*p* < 0.001). Some individual HI values in Eretria and Domokos were marginally higher than unity, but the average HI was below 1.0, indicating that in these areas the health risk was not significant. However, in the industrial area of Volos, HI values measured were significantly higher than unity, with the maximum value being as high as 4.99 (Figure 6; Table S22). 

## 4. Discussion

The soils in the sampled areas were all alkaline, with a medium content in CaCO_3_. The high pH values and the existence of CaCO_3_ are expected to have a protective role in binding PTEs in soil and in reducing their mobility towards plants.

Chromium was rather expectedly highly elevated in Eretria and Domokos, due to the history in Cr mining in these areas. Nickel, on the other hand, seems to be associated with Cr, i.e., the two metals are likely found in the same ore minerals excavated in the areas. This is plausible, given the frequently finding of Cr-Ni minerals: 8.34% of chromium ore bodies are cofound with Ni, the highest such percentage among co-existent metals with Cr ores [31]. It is also noteworthy that the relatively high concentrations of Ni in the steel factory (although approximately one order of magnitude lower than those in the other two study areas) is likely of natural origin, a not uncommon feature of the area, as recognized by Antoniadis et al. [21], who studied the same area. As for the relatively high Cr content in the steel factory soils, this must be associated to the industrial activities of that factory. Our findings concurred with Fry et al. [32], who studied the effect of metallurgical smelting activities on soil pollution in New Caledonia and reported similar soil concentrations for Fe, Cr, Mn, Ni, Pb, while Zn and Cu concentrations were noticeably lower. Pentari et al. [33] measured PTE concentrations in the vicinity of a coal mining site in Ptolemais, Greece, and found in soil Cd = 0.4, Co = 80, and Cr = 876 mg kg^−1^, comparable to the average total concentrations found in our study for the Eretria and Domokos mining sites. However, in the mining areas of Domokos and Eretria, higher values compared to the mining site of Ptolemais were reported for Ni (1271 vs. 655), while lower concentrations were measured in our work for Cu (10.06 vs. 44.4) and Pb (14.75 vs. 62.1; all units in mg kg^−1^). This shows that the studied areas were polluted above the baseline levels and could be characterized as moderately polluted (1 < PLI < 3; Figure 2; Table S8) [23]. Demková et al. [34] reported PLI values that were significantly higher than 3 in 80% of the soil samples they monitored in Slovakia. Similarly, Abliz et al. [35] assessed the pollution levels in the industrial zone of Xinjiang, China, and found that the average PLI of the topsoil samples were 4.8, being considerably higher than the average PLI value (1.61) measured in topsoil samples in the vicinity of the steel factory of Volos. These comparisons indicate that the studied areas were indeed very high in certain metals as caused by their history (mainly Cr and associated Ni in Eretria and Domokos), but the contamination was not related to other PTEs, thus exhibiting a relatively low PLI. Comparing our PLI values among studied areas, those of Eretria and Domokos were not statistically different (*p* = 0.582), while those from the industrial area of Volos (steel factory) were significantly lower (*p* < 0.001) than the other two. As for CF, some often-studied PTEs had CF near unity, indicating that their levels were close to their natural abundance concentrations: Cd was 1.79, Cu 1.43, Pb 1.26 and Zn 1.17 (Figure S1 and Table S7). Likewise, Demková et al. [34] measured the pollution levels at an abandoned mining site in Spiš, Slovakia, where mining activities were reportedly ceased early in the 21st century, and the reported CF values were 5.2 for Cd, 15.2 for Cu, 3.2 for Pb, and 12.1 for Zn, considerably higher than those found in our work. Contrary to that, the CF values of Co (0.9) and Cr (3.7) in that work were lower than those found here.

Concerning the PCA analysis, in Eretria in PC1 we identified an indication that with increasing distance from the mining site of Eretria, lower Ni, Co, Fe, and Cr concentrations were observed. Increased concentrations of Ni, Co, Fe and Cr in the vicinity of the mining area can be justified according to references relative to the chromite-rich parent matrix element content, reporting Ni concentrations ranging from 10 to 16,000, Co 180–1200, Fe^III^ 4.09–67.8%, and Cr 8950–11,270 mg kg^−1^ [11,15]. Thus, taking into consideration the PC1 loadings, combined with the results of different geological studies in the area, we can assume that Ni, Co, Fe and Cr concentrations were affected from the mining activity. The Cu weak-to-moderate negative association on PC1 indicates that increased Cu concentrations are observed with increasing distance from the mining site, similarly indicating anthropogenic sources. Cu and Zn were positively loaded on PC2 and their origin can thus be attributed to anthropogenic factors. Indeed, Cu and Zn are referred to as pollutants of anthropogenic origin for agricultural soils due to agrochemicals (mainly fertilizers and substances used for plant protection), although traffic and industrial emissions are also possible sources [36,37,38,39]. For PC3 we observed that the sources of these elements are different. In agricultural soils, Zn, Pb and Cu contamination can be attributed to atmospheric deposition of traffic and industrial emissions, while increased Cd concentrations in agricultural soils are likely attributed to fertilizer (especially phosphate) applications [39,40]. To our knowledge, Cd and Pb are not associated with chromite mining: Pb was only found in a limited number of chromite samples in New Zealand, being as low as 0.53 mg kg^−1^ [14], and the case of Cd is similar, with the only exception being a work by Mishra and Hazarika [41] reporting on greenockite (CdS) grains found onto chromite, a different case from our work. In Domokos, for PC1, based on the component loadings, Fe and Ni were identified with a derivation of different sources. Pb is attributed to anthropogenic factors (traffic and industrial emissions), while Fe and Ni loadings are affected from the chromite mining [11,15,39]. Relative to PC2, Mn and Co were highly associated, and for PC3, Cd and Zn were positively associated. Concerning PC2, Mn and Co positive loading can be attributed to the chromite mining activities due to the high Mn and Co concentrations found in chromites [11]. Positive loading of Cd and Zn for PC3 can be attributed to the application of fertilizers and pesticides [39,40,42]. Finally, loadings on PC4 indicate that with increasing distance from the mining site, Cr concentrations decreased, associating thus Cr levels to the mining site. Cr, Mn, Co and Ni in an earlier work in the same area were reported as being affected from the soil parent material and mainly from serpentinized ophiolites [20]. We also noticed that Cr, Co and Ni are positively associated to PC1 and Mn and Cu are positively associated to PC2, suggesting lithogenic sources. However, Cu is commonly found in agrochemicals used for plant protection and in fertilizers that are universally applied to cultivated fields [38,39]. Thus, Cu may be clustered with elements associated with natural sources. Industrial activities, including metal smelting, are considered important sources of Zn, Cd, and Pb. Moreover, Pb and Zn accumulation in the agricultural soils of this area was attributed to atmospheric deposition of traffic and industrial emissions [39,43]. For PC3, strong positive associations were noticed for Pb, Zn and moderate positive association for Cd, while weak-to-moderate negative loading was noticed for “distance” from the steel factory, indicating that the steel factory emissions may have affected soil Pb, Zn and Cd.

The high Zn levels in the wheat tissues in the steel factory samples can likely be attributed to the high availability of Zn (10.3%) as assessed by AB-DTPA (Table S18). In our study, the average AB-DTPA concentrations in the steel factory were higher than the reported values by Crispo et al. [44], who studied 20 UK cities. Tanzeem-ul-Haq et al. [45] studied Ni-affected soils, and reported that Ni availability in a soil of pH 7.03 was 3.02% of the total, comparable to the availability reported in our work.

The soil-to-plant transfer coefficient (TC) is a useful index showing the mobility of the studied PTEs to be absorbed by a test plant/crop. Hasnaoui et al. [46] measured PTE concentrations in native plants grown in a mining area, and reported a TC for Zn (in aerial tissues) equal to 190 (per mille), lower than our Zn TC values reported for Eretria (260) and Domokos (470). In the same industrial area of Volos as studied here, Antoniadis et al. [21] measured the TC values of maize and found that Mn in maize shoots were higher than the value in our work (95.3 vs. 52.1) and the Mn TC in grains lower than that in our work (6.58 vs. 47.5). As for Cr TC in shoots in the above-cited work, it was 4.79 and in grains 1.17, while in our work the values were much higher, 52.9 and 39.9, respectively. 

As for the health risk assessment, we found that the values for children were higher than unity for all three studied areas, indicating that soil ingestion may have negative health effects on children, which are more vulnerable [47]. Antoniadis et al. [21] monitored the industrial area of Volos and the average HI for soil ingestion was reported as equal to 3.24, considerably higher than the mean value found here (0.21). Similarly, Rinklebe et al. [48] assessed the health risk in the area of the floodplains across the central Elbe river, Germany; the risk associated to the soil ingestion of adults was 0.12 and of children 2.27, comparable to the values reported in our study concerning the steel factory (0.21 for adults and 1.97 for children). In another similar work, Antoniadis et al. [49] assessed the health risk in a highly contaminated site from mining activities in Germany, and the reported HI was 20.81. However, clearly HI values are dependent on PTE soil levels: in a work conducted in India, where PLI values were below 1.0, HI was similarly found very low [50]. Similar was the case reported by Zhang et al. [51] in China. Along these lines, Jimenez-Oyola et al. [52] in a gold mining-affected area assessed that health risk from soil ingestion was HI > 1, due to highly elevated PTE contents. The risk via soil ingestion is not by any means restricted to cultivated fields, but extends to dust ingestion on urban roads [53] and in indoor areas alike [54].

In similar cases as ours, concerning risk assessment via food consumption, food wheat flour associated health risk was found to be above unity in many cases, including a Pb smelting area [43]. On the other hand, in works reporting from routinely sampled food from the market, lower HI values were found [55]. Our HI was attributed mainly to the HQ values of Cr, Ni, Mn, and Zn. Thus, in the case of the consumption of flour deriving from grains produced in that area, health risk is considerable. Clearly food-induced HI is related to the PTE levels found in food, and also the amount consumed per capita [56,57]. Thus, rice-based dietary habits tend to lead to elevated risk when PTEs are found in this crop [58]. Similarly, wheat-based diets, frequently close to the dietary habits of the Western countries, can lead to elevated risk even if PTE contents in wheat are not necessarily extremely high [59]. In works performed in the Mediterranean, Di Bella et al. [60] assessed health risk after consumption of seabass in Sicily, and found that the risk related to fish PTEs was very low. In Spain, Fernandez-Landero et al. [61] found that the health risk deriving form As, Cd and Pb due to soil ingestion was likewise low. In Portugal, Cabral-Pinto et al. [62] found considerable risk in a study, which, similarly to ours, investigated edible crops around an industrial area. Austruy et al. [63] (for France), Osman et al. [64] (for Egypt), and Varol et al. [65] (for Turkey) are also examples of works related to health risk assessment linked to PTEs in soils and plants. 

## 5. Conclusions

In Eretria and Domokos, both known for their historic Cr mining activities, we found extremely high Cr soil concentrations, accompanied by highly elevated Ni, presumably also of the same origin. The PCA analysis confirmed the derivation of theses metals from the contamination epicenter, the chromite mines. In the steel factory area, there was not any particular highly offensive metal, but Ni and Cr were still high, Ni probably deriving from known natural geogenical origins and Cr from the factory activities. Due to the fact that soils were alkaline and calcareous, PTE mobility was rather limited, to the level that most of the studied PTEs were found below detection limits in wheat (both shoots and grains) and the soil-to-plant transfer coefficient was elevated only for the nutrient Zn (but Zn TC was still lower than 1.0). Health risk assessment via the soil ingestion pathway revealed an elevated problem for children, the most vulnerable group of people in all three studied areas, while for adults the risk was found to be acceptable. Likewise, the health risk via food (wheat grain) consumption was not of high risk for Eretria and Domokos, while it was found above unity (i.e., significant) for the steel factory area. We conclude that areas worldwide with a recorded history of harmful anthropogenic activities, even if currently ceased, especially when cultivated with crops destined for direct human consumption, such as wheat, must be thoroughly monitored and their contamination problems assessed concerning potential PTE-related health risks. To this end, we see this work as pivotal to other similar cases around the globe. However, we recognize the need for further in-depth work that would involve more plant species, native and cultivated alike, and for the investigation of the geochemical fractionation of the studied metals so that their behavior and fate may be assessed more thoroughly.

## Figures and Tables

**Table 1:** Descriptive statistics of selected soil properties of the three study areas, Eretria, Domokos and the Volos steel factory.

Eretria	pH	CaCO_3_	OC	Sand	Silt	Clay	Oxides
		%	%	%	%	%	mmol kg^−1^
10th-perc	6.77	0.12	0.89	33.48	19.04	6.00	26.97
50th-perc	7.08	0.27	1.70	50.80	30.40	20.00	54.00
Average	7.19	1.59	1.90	50.80	29.91	19.29	57.11
90th-perc	7.77	6.10	3.22	68.80	37.16	32.17	84.87
Domokos							
10th-perc	6.99	0.06	1.48	4.37	21.36	9.52	54.42
50th-perc	7.25	0.13	2.03	8.60	49.15	30.20	76.56
Average	7.34	0.34	2.51	26.24	43.75	30.01	78.36
90th-perc	7.77	1.12	3.50	67.80	64.28	45.60	106.36
Steel factory							
10th-perc	7.50	1.83	0.32	7.96	18.00	21.20	48.47
50th-perc	7.95	2.37	0.38	23.89	21.94	31.19	61.90
Average	8.14	4.10	1.25	34.83	28.39	35.60	74.19
90th-perc	8.10	5.15	1.25	34.43	28.71	36.87	76.19

OC: Organic carbon.

**Table 2:** Descriptive statistics of pseudo-total concentrations of potentially toxic elements (mg kg^−1^) in soil samples from Eretria, Domokos and the steel factory in the industrial area of Volos.

**Eretria**	**Cd**	**Co**	**Cr**	**Cu**	**Fe**	**Mn**	**Ni**	**Pb**	**Zn**
Min	0.27	20.24	199.6	3.32	29,865	616	76.9	11.5	35.6
10th-perc	0.27	25.54	244.7	5.49	32,741	740	243.7	11.5	38.0
50th-perc	0.41	74.94	687.2	9.71	42,633	969	1428.1	15.0	52.6
Average	0.51	68.38	705.2	11.56	42,690	1004	1227.5	15.1	53.8
90th-perc	0.72	115.14	1118.0	18.95	49,929	1387	2090.3	19.1	69.3
Max	0.82	123.62	1272.2	29.30	52,178	1676	2236.6	23.1	109.3
Skewness	0.40	0.00	0.00	1.29	−0.47	1.12	−0.25	0.64	1.93
**Domokos**	**Cd**	**Co**	**Cr**	**Cu**	**Fe**	**Mn**	**Ni**	**Pb**	**Zn**
Min	0.41	36.65	362.1	4.13	30,169	609	330.5	11.5	21.8
10th-perc	0.41	52.35	590.6	6.54	32,199	874	900.5	11.8	33.6
50th-perc	0.41	82.87	712.7	8.19	42,845	1100	1339.6	13.8	42.5
Average	0.45	84.05	777.5	8.57	41,325	1215	1315.1	14.4	44.6
90th-perc	0.56	109.40	925.3	11.27	45,873	1664	1788.1	16.4	52.3
Max	0.68	137.30	1560.0	13.47	47,193	1957	1811.7	20.7	90.7
Skewness	2.08	0.05	1.53	0.39	−1.39	0.62	−1.06	0.71	1.90
**Steel Factory**	**Cd**	**Co**	**Cr**	**Cu**	**Fe**	**Mn**	**Ni**	**Pb**	**Zn**
Min	0.00	12.03	67.8	12.08	17,992	431	53.4	0.0	48.0
10th-perc	0.00	15.40	141.7	14.51	23,529	504	80.6	0.0	50.2
50th-perc	0.17	25.54	350.3	19.75	34,112	632	258.2	0.0	82.6
Average	0.18	26.40	359.4	21.43	32,377	648	257.6	3.4	86.1
90th-perc	0.32	38.49	658.7	30.05	40,522	804	467.5	10.2	116.8
Max	0.52	47.71	712.8	35.22	44,307	829	548.4	25.1	215.2
Skewness	0.71	0.45	0.35	0.54	−0.22	0.05	0.43	2.21	1.84
BG	0.41	11.3	59.5	38.9	---	480	29	27	70
TAV	20	100	450	500	---	---	150	300	1500
MAC	5	50	200	150	---	---	60	300	300

BG = background content of trace elements in earth crust, TAV = trigger action values, MAC = maximum allowable concentrations.

**Table 3:** Transfer coefficients (concentration in plant tissues over total concentration in soil) of potentially toxic elements in Eretria, Domokos and the steel factory—industrial area of Volos. Values are reported magnified by a factor of 10^3^ for clarity. The comparisons are made among elements for each plant part (grains and shoots), and between plant parts for each element. Different letters within columns denote significance for each element at *p* < 0.05.

		Shoots	Grains	Significance	*p*-Value
Eretria	Cd	nd	nd	---	---
	Co	nd	nd	---	---
	Cr	nd	nd	---	---
	Cu	nd	nd	---	---
	Fe	0.84	nd	---	---
	Mn	9.96	4.32	ns	0.115
	Ni	nd	nd	---	---
	Pb	nd	nd	---	---
	Zn	290.90	233.84	ns	0.721
Domokos	Cd	nd	nd	---	---
	Co	nd	nd	---	---
	Cr	nd	nd	---	---
	Cu	nd	nd	---	---
	Fe	0.59	nd	---	---
	Mn	8.35	11.20	***	<0.001
	Ni	nd	nd	---	---
	Pb	nd	nd	---	---
	Zn	415.60	570.15	***	<0.001
Steel factory	Cd	nd	nd	--	--
	Co	nd	nd	--	--
	Cr	52.93	39.94	**	<0.01
	Cu	407.96	442.21	ns	0.517
	Fe	nd	nd	--	--
	Mn	52.10	47.50	ns	0.677
	Ni	nd	nd	--	--
	Pb	nd	nd	--	--
	Zn	516.89	556.33	ns	0.208

** = significant at the level of *p* < 0.01; *** = significant at the level of *p* < 0.001; ns = nonsignificant; nd = not determined, due to the fact that either the numerator or the denominator of the formula of TC was zero; -- = not calculated due to lack of data.

## Data Availability

Raw data that support the findings of this study available on request.

## Supplementary Materials

The following are available online at https://www.mdpi.com/article/10.3390/toxics9110293/s1, Figure S1: Contamination factor (CF, unitless) boxplot for the soil samples obtained from (a) Eretria, (b) Domokos and (c) the industrial area of Volos (near the steel factory), Figure S2: Scree plot of PCs and % of variance explained from the components, Eretria dataset, Figure S3: Scree plot of PCs and % of variance explained from the components, Domokos dataset, Figure S4: Scree plot of PCs and % of variance explained from the components, steel factory, Volos dataset, Figure S5: Hazard quotient (of the soil ingestion pathway), in Eretria for adults (a) and children (b), in Domokos for adults (c) and children (d) and in the Volos steel factory for adults (e) and children (f), Table S1: Sampling positions in Eretria and soil physicochemical characteristics, Table S2: Sampling positions in Domokos and soil physicochemical characteristics, Table S3: Sampling coordinates of Steel factory sampling sites (Volos industrial area) and soil physicochemical characteristics, Table S4: Pseudo-total concentrations of potentially toxic elements (mg kg^−1^) in soil samples from Eretria, Domokos and steel factory (industrial area of Volos), Table S5: Equations of total potential trace element concentrations as a function of the pollution source distance from the sampling site, Table S6: Pearson correlation coefficient values for the total element concentrations in the three studied areas, Table S7: Descriptive statistics of CF values in Eretria, Domokos and steel factory (industrial area of Volos), Table S8: Descriptive statistics for Pollution Load Index (PLI) for the studied areas, Table S9: Pearson correlation of PLI index values with distance from suspected pollution source, Table S10: Varimax rotated factor loading matrix for Eretria sampling area, Table S11: Rotated component matrix of principal component analysis for heavy metals, component correlation matrix, KMO and Bartlett’s test for Eretria sampling sites, Table S12: Varimax rotated factor loading matrix for Domokos sampling sites, Table S13: Rotated component matrix of principal component analysis for heavy metals, component correlation matrix, KMO and Bartlett’s test for Domokos sampling sites, Table S14: Varimax rotated factor loading matrix for steel factor sampling sites; Table S15: Rotated component matrix of principal component analysis for heavy metals, component correlation matrix, KMO and Bartlett’s test for steel factory sampling sites, Table S16: Potentially toxic elements in cereal shoot tissues (mg kg^−1^) in Eretria, Domokos and steel factory (industrial area of Volos), Table S17: Potentially toxic elements in cereal grains (mg kg^−1^) in Eretria, Domokos and steel factory (industrial area of Volos), Table S18: Descriptive statistics of potentially toxic elements extracted using AB-DTPA (ammonium bicarbonate-diethylene-triamine pentaacetic acid, mg kg^−1^) and percentage of availability of AB-DTPA-extractable elements relative to the pseudo-total elements, Table S19: Duncan tests for HQ soil ingestion in the three studied areas: adults (a), children (b), Table S20: Duncan tests for Eretria HQ (soil ingestion) adults (a) and children (b), Domokos HQ adults (c) and children (d) and steel factory (Volos) HQ adults (e) and children (f), Table S21: Hazard Index (HI) for soil ingestion in the three studied areas, Table S22: Hazard Index for humans from wheat grain consumption.
