# Supplementary material for: Monitoring Potentially Toxic Element Pollution in Three Wheat-Grown Areas with a Long History of Industrial Activity and Assessment of Their Effect on Human Health in Central Greece
Source: Toxics. 2021 Nov 4;9(11):293. doi: 10.3390/toxics9110293 (PMC8624685; doi:10.3390/toxics9110293)
Supplement: Supplementary file 1 [file toxics-09-00293-s001.zip › toxics-1437551-done.pdf]

toxics

MDPI

Supplementary Materials: Monitoring Potentially Toxic Element Pollution in Three Wheat-Grown Areas with a Long History of Industrial Activity and Assessment of Their Effect on Human Health in Central Greece

Georgios Thalassinos and Vasileios Antoniadis

1. Introduction

The spatial distribution of heavy metals in monitoring programs results in datasets that are complex to interpret due to the correlation between the studied variables. Principal Component Analysis (PCA) is a multivariate statistical method used to determine differences between the initially measured variables. PCA explains the quantitative relation between the variables of the original dataset by transforming them into a new dataset of new variables, called Principal Components (PCs), that summarize the features of the originally measured variables and are linearly independent [1]. PCA is commonly used in environmental data analysis to reduce the number of variables through the calculation of PCs that incorporate a significant part of the original data variability. In this respect, PCs containing a significant part of the initial variance facilitate the discrimination of any correlation between the measured variables. PCA ultimately leads to variable classification according to the factor loading for each principal component, and the results can be used to identify the sources of heavy metals in soil samples. Heavy metals of a common source cluster on high loading values, thus, their correlation can be identified [1–2].

2. Materials and Methods

PCA was performed to identify the potential sources of heavy metals. The number of PCs in PCA was determined using the Eigenvalue—one criterion: any component of Eigenvalue greater than unity incorporate a larger amount of variance compared to the variables of the original dataset. Ultimately, PCA reduces the number of variables that describe the variance and correlations between the measured parameters into a minimal number of linearly independent variables, named principal components (PCs). In this respect, a major part of the variance noticed in the original dataset is extracted using a limited number of principal components that facilitate the interpretation of the results [3–5]. Factor loading of the measured parameters on PCs can be classified as strong when > 0.70, moderate for loadings ranging from 0.70–0.50 and weak for values < 0.50 [5]. Varimax (variance maximizing) rotation of the original variable space was used to maximize the variance extracted from the principal components [6].

3. Results and Discussion

3.1. Potentially Toxic Elements in Relation to the Distance from the Contamination Source

Assuming a certain point source of pollution in the study areas, with the epicenter being the specific industrial activities, we developed equations that describe the total element concentrations as a function of the distance from the suspected pollution sources (Table S5); the Pearson correlation coefficient values indicated that, for Eretria, Ni, Co, Fe, Cr and Mn significantly decreased with distance from the mining sites, thus exhibiting a strong association to this anthropogenic activity. The linear equations that estimated the PTE concentrations as a function of the distance from the source had the following coefficients of determination: (R^2): 0.767 (for Ni), 0.619 (Co), 0.536 (Fe), 0.393 (Cr), 0.2739 (Cd) and 0.258 (Mn), all significant at the level of $p < 0.05$ (Table S5), with Pearson correlation

coefficients (r) being –0.876 (at $p < 0.001$) for Ni, –0.783 ($p < 0.001$) for Co, –0.733 ($p < 0.001$) for Fe, –0.626 ($p = 0.005$) for Cr and –0.508 ($p = 0.031$) for Mn (Table S6). In Domokos, the coefficients of determination were low, indicating that the concentrations of most of the elements fluctuated in an inconsistent pattern; thus, there was no causal effect that could be traced specifically to the ceased mining activity. However, Cr was indeed significantly negatively associated with distance (it decreased with increasing distance), thus confirming the fact that this specific element was the only one to be likely released from the mining activity. The coefficient of determination for Cr was $R^2 = 0.217$, and the Pearson correlation coefficient (r) was –0.466 (although seemingly low, still significant at $p = 0.038$; Table S5). As for the steel factory area of Volos, the coefficients of determination were significant, although relatively low, for Pb and Zn, a finding indicating that they are likely associated to the steel processing activities of the factory (Pb $r = -0.422$ at $p = 0.020$; Zn $r = -0.386$ at $p = 0.035$). As for the association between metals, in Eretria, Cr was correlated with Fe, Ni and Pb, a finding confirming the previously reported data (Table S6). As for Domokos, Cr had apparently no significant correlation to any of the measured elements. In the steel factory area, significant positive correlations were found for Cr vs. Co, Fe vs. Ni, Co vs. Cr, Fe vs. Ni and Fe vs. Co, Cr, Cu, Mn and Ni. The above results are in accordance with the findings of Olowoyo-Odiwe-Mkolo [7], where a highly significant positive correlation of Fe and Cr with Ni was reported, signifying a common source and mineral association and/or soil fractionation and chemical behavior. In addition, we examined the dependence of PLI with distance from the suspected contamination epicenter. In Eretria, the PLI values significantly decreased with distance ($p = 0.002$), while those in Domokos and in the industrial area of Volos (steel factory) were not ($p = 0.220$ and $p = 0.695$, respectively) (Table S9).

3.2. Contamination Factor of the Studied Potentially Toxic Elements

Contamination factor (CF) is a useful index functioning as a "unitless" concentration that allows the comparison among elements. Our data illustrate that in the three studied areas, as was expected, Ni, Cr and Co exerted a major effect on the pollution load (Figure. S2, Table S7). The values (average across studied areas: Ni CF = 33.19; Cr CF = 10.32) were high in comparison to other findings in the literature. Other often-studied PTEs had CFs near unity, indicating that their levels were close to their natural abundance concentrations: Cd was 1.79, Cu 1.43, Pb 1.26 and Zn 1.17, values still higher than those reported in the previously cited work (Cd = 0.92, Cu = 0.36, Pb = 0.41 and Zn = 0.88). In addition, Demková [8] measured the pollution levels at an abandoned mining site in Spiš, Slovakia, where mining activities were reportedly ceased early in the 21st century: The reported CF values were 5.2 for Cd, 15.2 for Cu, 3.2 for Pb and 12.1 for Zn, considerably higher than those found in our work. Contrary to that, the CF values of Co (0.9) and Cr (3.7) in that work were lower than those found here.

3.3. The Necessity for Principal Component Analysis

For a dataset to be suitable for PCA analysis, the Kaiser–Meyer–Olkin Measure of Sampling Adequacy (KMO) values are required to be greater than 0.5, and the result from Bartlett's Test of Sphericity must be statistically significant ($p < 0.05$). More specifically, in this work, the KMO values (0.553 in Eretria, 0.514 in Domokos and 0.653 in the steel factory dataset) and the statistically significant results of Bartlett's Test of Sphericity ($p < 0.001$) for the three studies (Tables S10–15) signified that the variables in each dataset were associated and not orthogonal and that a PCA analysis would give credible results [9,5]. Furthermore, the correlation values among the extracted PCs were, on average, 0.1233 for Eretria, 0.0683 for Domokos and –0.1637 for the steel factory PCs (Figure S3).

3.4. Available PTE Concentrations (AB-DTPA)

For the studied areas, the potentially phyto-available concentrations were measured with AB-DTPA (Table S18). In Eretria, the Cd, Cr and Pb AB-DTPA concentrations were below the detection limits, while the percentage of availability (i.e., the DTPA levels over the corresponding aqua regia levels) of Co, Cu, Fe, Mn, Ni and Zn ranged from 0.84 to 7.80% (Table S18). The available PTE concentrations of Domokos were similar to those of Eretria, with the exception of Cu, where its availability was higher. As for the steel factory of Volos, Pb, Cd, Cu and Zn had higher availability percentage than expected: They equaled 139.0%, 56.7%, 25.2% and 10.3%, respectively, indicating anthropogenic sources of pollution. However, in those PTEs where high pseudo-total concentrations were measured, the AB-DTPA levels were relatively low, with the percentages of availability ranging from 0.03% for Cr to 3.22% for Ni; this indicates that these elements may be of geogenic origin. In our study, the average AB-DTPA concentrations in the steel factory were for Ni = 6.75, for Pb = 5.30, for Cu = 5.28, for Zn = 10.45, for Cr = 0.14 and for Cd = 0.12 (all units in mg kg-1), which were higher than the reported values by Crispo [10], who reported from 20 UK cities Ni = 0.068, Pb = 0.023, Cu=0.18, Zn = 4.73, Cr = 0.1 and Cd = 0.005. Tanzeem-ul-Haq [11] studied Ni-affected soils and reported that Ni availability in a soil of pH 7.03 was 3.02% of the total, comparable to the availability reported in our work.

3.5. HQ Values for Soil Particle Ingestion

HQ values for soil ingestion both for adults and children were significantly different in the three studied areas in the order of Domokos > Eretria > steel factory ($p < 0.001$) (Figure. S5; Table S19–20). In all studied areas, Co, Fe and Ni had significantly higher HQ values than the rest of the PTEs ($p < 0.001$), while for Mn, Pb, Cd, Cr, Cu and Zn, minimal values were observed (Table S13). This shows that Cr, the most elevated and problematic of all studied PTEs in soil, is the least offensive concerning health risk, probably due to the fact that it is a nutrient for humans, and thus its RfDo is comparatively high (1500), while that of Co is only 0.3 (units in μg day^{-1} kg^{1} BW).

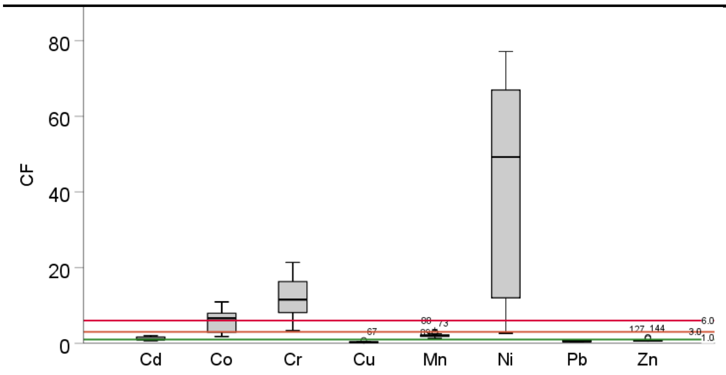

(a)

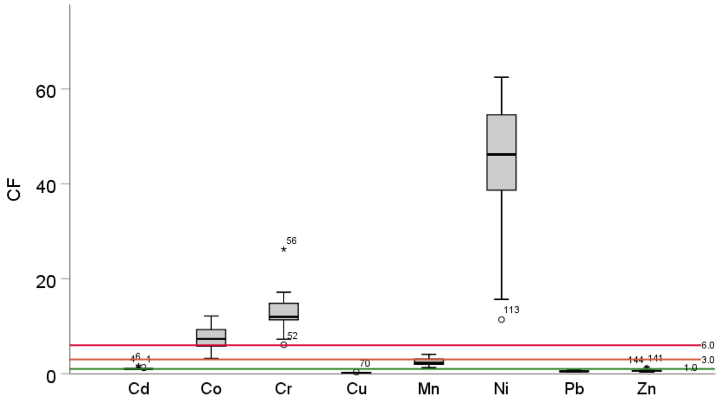

(b)

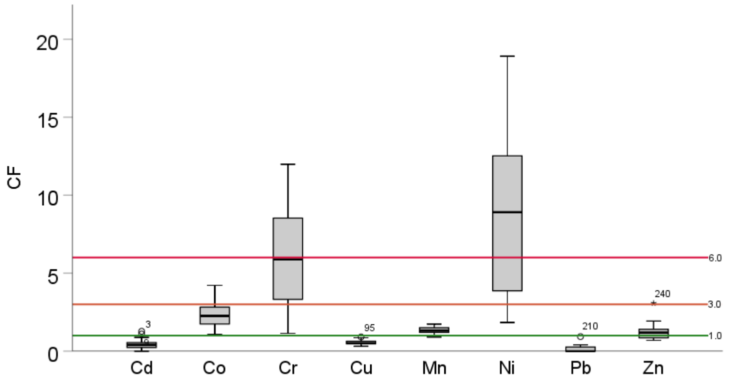

(c)

Figure S1. Contamination factor (CF, unitless) boxplot for the soil samples obtained from (**a**) Eretria, (**b**) Domokos and (**c**) the industrial area of Volos (near the steel factory).

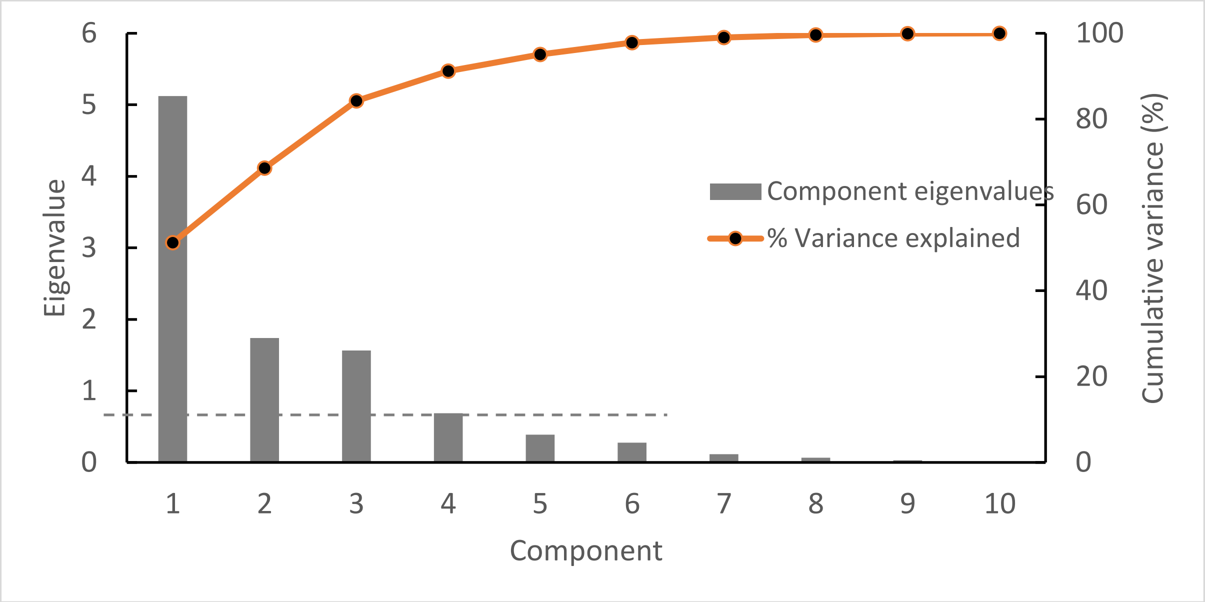

Figure S2. Scree plot of PCs and % of variance explained from the components, Eretria dataset.

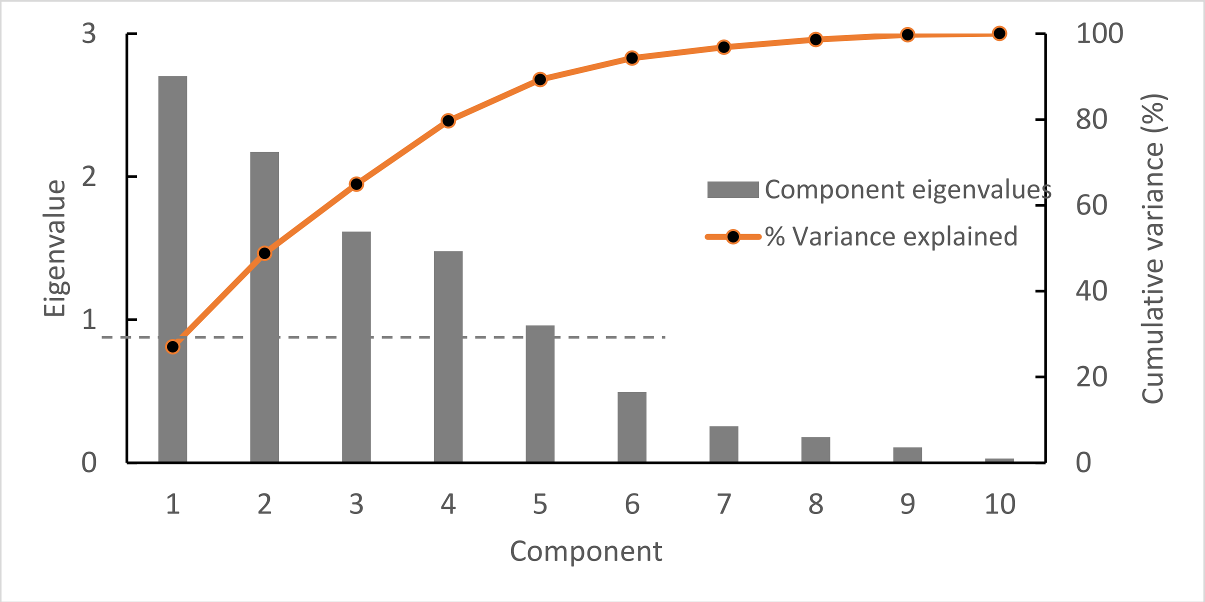

Figure S3. Scree plot of PCs and % of variance explained from the components, Domokos dataset.

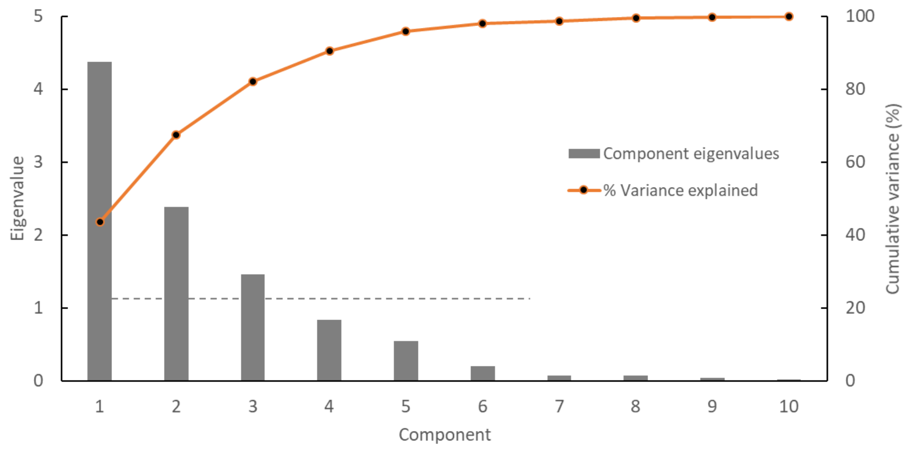

Figure S4. Scree plot of PCs and % of variance explained from the components, steel factory, Volos dataset.

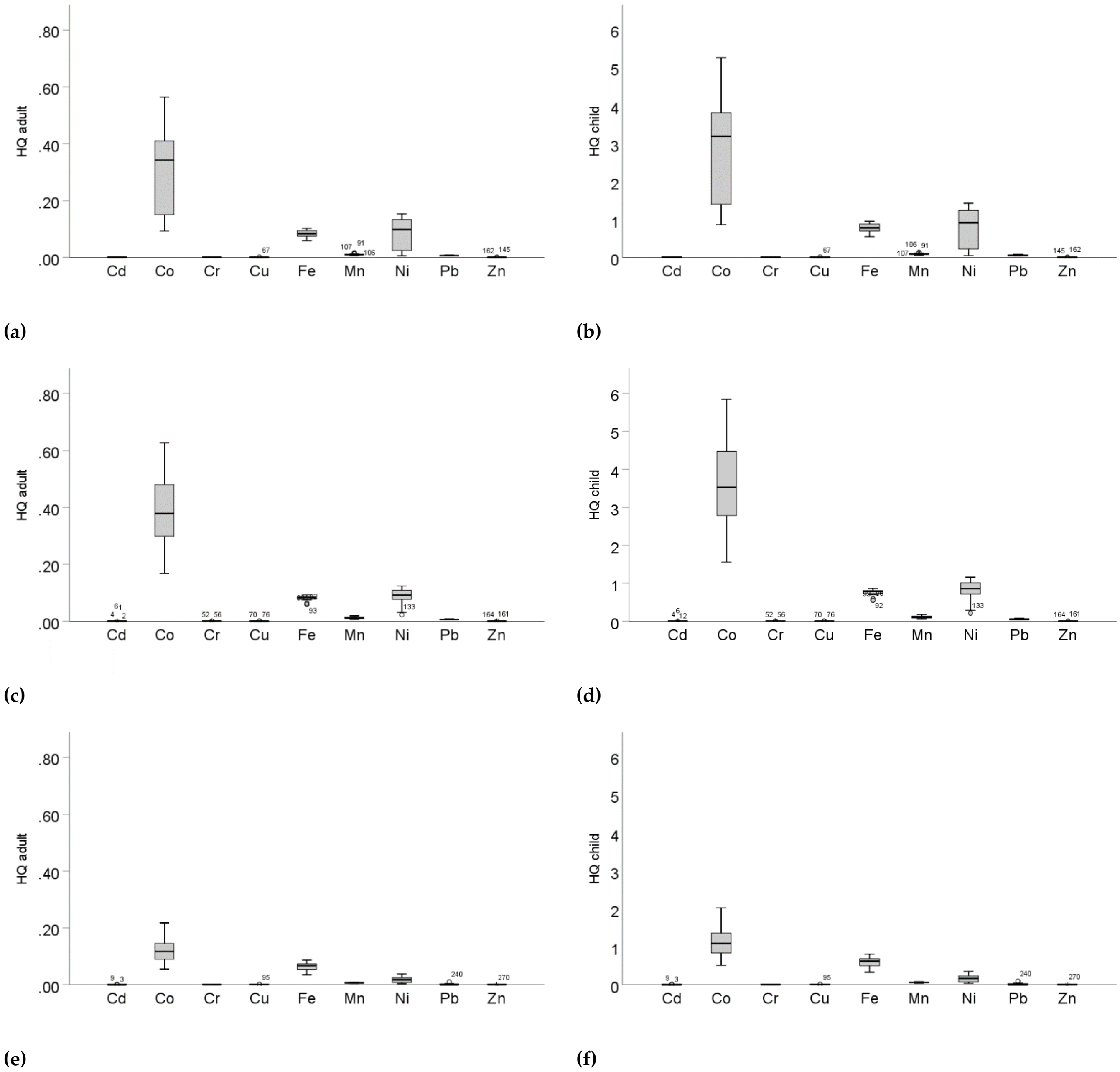

Figure S5. Hazard quotient (of the soil ingestion pathway), in Eretria for adults (**a**) and children (**b**), in Domokos for adults (**c**) and children (**d**) and in the Volos steel factory for adults (**e**) and children (**f**).

Table S1. Sampling positions in Eretria and soil physicochemical characteristics.

Eretria	N@39⁰	E@22⁰	Soil type[a]	Distance[b] m	pH	$CaCO_3$ %	OC %	Sand %	Clay %	Oxides mmol kg^{-1}
**1**	18′01.6″	37′30.0″	LP	167	6.38	0.21	2.42	51.2	20.0	80.60
**2**	17′58,7″	37′36,2″	LP	279	6.72	0.31	2.89	77.2	8.0	68.16
**3**	17′59,2″	37′49,8″	LP	558	6.91	0.21	1.87	55.3	21.1	58.14
**4**	17′54,3″	38′00,3″	LP	847	6.79	0.18	1.79	60.0	20.0	65.59
**5**	17′49,1″	38′11,5″	LP	1153	6.93	0.29	1.56	54.0	22.0	65.29
**6**	17′47,4″	38′23,0″	LP	1430	7.07	0.21	1.76	30.3	33.3	49.86
**7**	17′44,9″	38′22,4″	LP	1443	6.86	0.25	1.64	28.0	34.0	49.50
**8**	18′19,0″	37′14,0″	LP	554	7.09	0.23	1.21	65.2	12.0	69.20
**9**	18′19,5″	37′16,8″	LP	515	7.63	5.70	1.25	77.2	6.0	27.14
**10**	18′45,8″	36′56,6	CL	1468	7.76	7.65	0.78	47.2	18.0	17.53
**11**	18′46,5″	37′08,0	CL	1368	7.84	4.27	1.17	43.2	20.0	26.57
**12**	18′59,9″	36′52,8″	CL	1896	7.78	0.45	0.94	41.6	5.6	37.45
**13**	18′37,4″	36′52,8″	CL	1307	7.52	0.34	0.78	55.2	14.0	44.88
**14**	18′28,0″	37′11,6″	LP	822	7.69	7.03	1.17	65.2	6.0	44.79
**15**	17′47,7″	37′20,5″	LP	565	7.25	0.12	3.35	34.8	31.7	81.71
**16**	17′41,5″	37′25,7″	LP	724	6.94	0.04	3.16	37.2	30.0	104.53
**17**	17′42,4″	37′28,8″	LP	697	6.80	0.12	3.55	41.2	28.8	92.22
**18**	17′46,6″	37′06,6″	LP	740	7.40	1.03	2.89	50.4	16.8	44.88

a. LP: Leptosols; CL: Calcisols; b. Distance in m from the suspected contamination point. N@39⁰: All sampling points are situated at 39 degrees North. E@22⁰: All sampling points are situated at 22 degrees East.

Table S2. Sampling positions in Domokos and soil physicochemical characteristics.

**Domokos**	**N@39⁰**	**E@22⁰**	**Soil**	**Distance[b] m**	**pH**	**$CaCO_3$ %**	**OC %**	**Sand %**	**Clay %**	**Oxides mmol**
**1**	4′38.8″	19′08.8″	VR	296	7.51	1.68	4.80	9.2	43.7	68.22
**2**	4′48.1″	19′37.3″	VR	524	7.16	0.13	3.35	12.0	44.9	117.21
**3**	4′56.4″	19′55.7″	CM	1015	7.25	0.30	2.81	6.8	25.3	83.14
**4**	5′25.3″	19′42.9″	CM	1378	6.84	0.25	6.32	12.4	28.8	94.10
**5**	5′14.6″	19′47.9″	CM	1171	7.09	0.15	2.65	4.4	22.8	102.93
**6**	5′6.0″	19′46.7″	CM	965	6.97	0.08	3.08	4.4	28.8	68.49
**7**	5′13.0″	20′13.5″	CM	1634	7.08	0.12	2.81	6.4	27.6	109.93
**8**	5′05,6″	20′13,4″	CM	1487	7.16	0.06	2.73	4.4	35.6	82.88
**9**	4′38.4″	18′48.9″	VR	690	7.53	0.06	1.95	3.6	44.0	105.96
**10**	4′38.9″	18′37.1″	CM	955	7.64	0.10	2.03	4.5	14.3	49.48
**11**	4′32.2″	18′39.1″	VR	983	7.87	0.16	1.87	5.6	35.7	85.39
**12**	4′18.5″	18′43.6″	VR	1157	7.96	1.07	1.83	8.0	48.0	62.23
**13**	4′11.7″	18′23.0″	VR	1662	7.76	1.60	2.65	4.2	57.3	41.11
**14**	4′52.5″	18′43.9″	CM	787	7.17	0.30	1.83	59.7	13.4	57.37
**15**	4′52.7″	18′52.4″	VR	594	7.35	0.06	1.79	67.6	39.6	69.56
**16**	4′47.9″	18′58.4″	VR	420	7.73	0.41	0.90	49.6	31.6	54.97
**17**	5′02.6″	18′44.3″	CM	905	7.24	0.08	1.44	55.6	21.6	88.86
**18**	5′02.6″	18′50.6″	VR	786	6.99	0.05	1.83	61.6	42.8	70.24
**19**	5′3.7″	18′56.8″	VR	699	7.12	0.13	2.03	75.2	45.6	86.47
**20**	4′57.3″	18′58.1″	VR	540	7.38	0.08	1.48	69.6	38.8	68.69

a. CM: Cambisols; VR: Vertisols; b. Distance in m from the suspected contamination point. N@39⁰: All sampling points are situated at 39 degrees North. E@22⁰: All sampling points are situated at 22 degrees East.

Table S3. Sampling coordinates of Steel factory sampling sites (Volos industrial area) and soil physicochemical characteristics.

Steel factory	N@39⁰	E@22⁰	Soil	Distance[b] m	pH	$CaCO_3$ %	OC %	Sand %	Clay %	Oxides mmol
**1**	23′03.7′′	49′00.4′′	CM	913	8.27	5.53	1.58	32.01	33.19	65.22
**2**	23′03.7′′	49′04.2′′	CM	1000	8.09	6.17	2.13	34.84	39.18	74.89
**3**	23′02.4′′	49′12.4′′	CM	1194	8.17	17.67	1.01	34.41	35.99	89.11
**4**	23′12.1′′	47′02.6′′	CM	1905	8.04	3.15	0.69	7.96	49.22	70.33
**5**	23′14.2′′	46′37.8′′	CM	2525	7.96	2.30	1.80	19.95	41.22	73.90
**6**	23′12.3′′	46′15.7′′	CM	3045	7.5	2.54	0.85	38.38	36.01	66.68
**7**	23′10.3′′	46′49.8′′	CM	2225	8.35	2.66	0.52	41.95	33.23	62.49
**8**	23′09.6′′	46′58.1′′	CM	2023	8.23	5.20	0.78	15.98	47.21	84.77
**9**	23′06.7′′	47′36.6′′	CM	1104	7.91	2.68	2.03	34.81	33.19	74.47
**10**	23′05.6′′	47′40.1′′	CM	1022	8.21	2.38	0.34	25.97	43.22	93.19
**11**	23′02.1′′	47′38.1′′	CM	1076	8.25	3.94	1.65	24.74	45.24	102.58
**12**	23′01.1′′	47′44.7′′	CM	925	8.16	1.96	0.32	26.34	42.03	123.78
**13**	22′52.9′′	47′24.0′′	CM	1465	8.06	2.98	1.80	38.85	33.17	65.41
**14**	22′51.2′′	47′27.5′′	CM	1405	8.13	5.80	0.52	32.36	34.02	69.23
**15**	22′49.9′′	47′31.5′′	CM	1334	8.07	4.26	2.10	34.77	35.21	75.94
**16**	22′45.7′′	47′15.9′′	CM	1728	7.92	7.00	0.79	38.44	33.98	55.54
**17**	22′42.4′′	47′21.8′′	CM	1646	8.19	2.88	0.38	40.05	31.18	65.54
**18**	22′47.8′′	47′37.2′′	CM	1249	8.06	3.18	1.72	36.83	37.19	79.10
**19**	22′50.3′′	47′45.5′′	CM	1020	8.12	5.61	1.95	42.02	33.19	79.17
**20**	22′51.9′′	47′54.0′′	CM	822	8.01	2.81	0.33	24.32	44.04	86.30
**21**	22′54.3′′	48′01.9	CM	617	7.98	6.96	1.19	40.84	37.18	72.83
**22**	22′56.5′′	48′09.9′′	CM	425	8.24	5.57	0.73	48.49	29.95	67.14
**23**	22′57.5′′	48′17.0′′	CM	309	8.16	2.91	2.33	40.81	31.19	81.72
**24**	22′58.3′′	48′22.1′′	CM	240	8.15	4.84	1.76	60.79	21.20	56.56
**25**	22′58.7′′	48′28.5′′	CM	265	8.17	1.83	0.57	34.39	36.01	96.26
**26**	22′56.9′′	48′33.9′′	CM	384	8.09	23.10	1.31	26.46	45.96	71.43
**27**	22′54.4′′	48′31.5′′	CM	427	8.17	5.23	2.13	28.03	47.18	92.97
**28**	22′49.1′′	48′26.6′′	CM	551	7.95	5.22	1.42	42.35	32.03	63.50
**29**	22′53.4′′	47.58.8′′	CM	698	8.2	5.52	0.45	48.82	31.19	77.22
**30**	23′04.1′′	48′23.0′′	CM	58	8.14	2.68	2.44	36.83	33.19	48.47

a. CM: Cambisols; b. Distance in m from the suspected contamination point. N@39⁰: All sampling points are situated at 39 degrees North. E@22⁰: All sampling points are situated at 22 degrees East.

Table S4. Pseudo-total concentrations of potentially toxic elements (mg kg^{-1}) in soil samples from Eretria, Domokos and steel factory (industrial area of Volos).

Eretria	Cd	Co	Cr	Cu	Fe	Mn	Ni	Pb	Zn
**Min**	0.27	20.24	199.6	3.32	29865	616	76.9	11.5	35.6
**10th perc**	0.27	25.54	244.7	5.49	32741	740	243.7	11.5	38.0
**50th perc**	0.41	74.94	687.2	9.71	42633	969	1428.1	15.0	52.6
**Average**	0.51	68.38	705.2	11.56	42690	1004	1227.5	15.1	53.8
**90th perc**	0.72	115.14	1118.0	18.95	49929	1387	2090.3	19.1	69.3
**Max**	0.82	123.62	1272.2	29.30	52178	1676	2236.6	23.1	109.3
**Skewness**	0.40	0.00	0.00	1.29	-0.47	1.12	-0.25	0.64	1.93
**Domokos**	**Cd**	**Co**	**Cr**	**Cu**	**Fe**	**Mn**	**Ni**	**Pb**	**Zn**
**Min**	0.41	36.65	362.1	4.13	30169	609	330.5	11.5	21.8
**10th perc**	0.41	52.35	590.6	6.54	32199	874	900.5	11.8	33.6
**50th perc**	0.41	82.87	712.7	8.19	42845	1100	1339.6	13.8	42.5
**Average**	0.45	84.05	777.5	8.57	41325	1215	1315.1	14.4	44.6
**90th perc**	0.56	109.40	925.3	11.27	45873	1664	1788.1	16.4	52.3
**Max**	0.68	137.30	1560.0	13.47	47193	1957	1811.7	20.7	90.7
**Skewness**	2.08	0.05	1.53	0.39	-1.39	0.62	-1.06	0.71	1.90
**Steel Factory**	**Cd**	**Co**	**Cr**	**Cu**	**Fe**	**Mn**	**Ni**	**Pb**	**Zn**
**Min**	0.00	12.03	67.8	12.08	17992	431	53.4	0.0	48.0
**10th perc**	0.00	15.40	141.7	14.51	23529	504	80.6	0.0	50.2
**50th perc**	0.17	25.54	350.3	19.75	34112	632	258.2	0.0	82.6
**Average**	0.18	26.40	359.4	21.43	32377	648	257.6	3.4	86.1
**90th perc**	0.32	38.49	658.7	30.05	40522	804	467.5	10.2	116.8
**Max**	0.52	47.71	712.8	35.22	44307	829	548.4	25.1	215.2
**Skewness**	0.71	0.45	0.35	0.54	-0.22	0.05	0.43	2.21	1.84
**BG**	0.41	11.3	59.5	38.9	---	480	29	27	70
**TAV**	20	100	450	500	---	---	150	300	1500
**MAC**	5	50	200	150	---	---	60	300	300

BG=Background content of trace elements in earth crust, TAV=Trigger Action Values, MAC= Maximum Allowable Concentrations

Table S5. Equations of total potential trace element concentrations as a function of the pollution source distance from the sampling site.

**Eretria**	**Linear**	**Logarithmic**	**Exponential**
Cd	-0.0002x+0.69 (R^2 = 0.2739)	-0.144ln(x)+1.472 (R^2 = 0.2438)	$0.7255e^{-0.0004x}$ (R^2 = 0.2623)
Co	-0.0541x+120.2 (R^2 = 0.619)	-37.25ln(x)+318.3 (R^2 = 0.5074)	$153.4e^{-0.001x}$ (R^2 = 0.555)
Cr	-0.428x+1098 (R^2 = 0.393)	-235.3ln(x)+2273.5 (R^2 = 0.204)	$1245.8e^{-0.0008x}$ (R^2 = 0.316)
Cu	+0.0033x+8.51 (R^2 =0.059)	+1.901ln(x)-1.092 (R^2 = 0.033)	$7.095e^{0.0004x}$ (R^2 = 0.528)
Fe	-10.43x+52270 (R^2 = 0.536)	-7371ln(x)+91824 (R^2 = 0.4609)	$53367e^{-0.0003x}$ (R^2 = 0.527)
Mn	-0.2819x+1263 (R^2 = 0.258)	-200.8ln(x)+2342 (R^2 = 0.225)	$1258.7e^{-0.0003x}$ (R^2 = 0.2547)
Ni	-1.4435x+2553 (R^2 = 0.767)	-1045ln(x)+8195 (R^2 = 0.692)	$4388.1e^{-0.002x}$ (R^2 = 0.673)
Pb	-0.002x+16.954 (R^2 = 0.068)	-1.323ln(x)+23.9 (R^2 = 0.0514)	$16.57e^{-0.001x}$ (R^2 = 0.068)
Zn	-0.0137x+66.42 (R^2 = 0.124)	-37.25ln(x)+318 (R^2 = 0.5074)	$61.56e^{-0.0002x}$ (R^2 = 0.1313)
Domokos	Linear equation	Logarithmic equation	Exponential equation
Cd	-0.00005x+0.499 (R^2 = 0.057)	-0.064ln(x)+0.8775 (R^2 = 0.106)	$0.4862e^{-0.0001x}$ (R^2 = 0.059)
Co	-0.0192x+101.96 (R^2 = 0.092)	-15.06ln(x)+185.65 (R^2 = 0.077)	$102.7e^{-0.0003x}$ (R^2 = 0.089)
Cr	-0.2896x + 1047 (R^2 = 0.217)	-244.3ln(x) + 2425 (R^2 = 0.211)	$1047e^{-0.0004x}$ (R^2 = 0.224)
Cu	-0.00006x + 8.6 (R^2 = 0.011)	0.038ln(x) + 8.32 (R^2 = 0.0007)	$8.073e^{0.00003x}$ (R^2 = 0.0001)
Fe	-1.292x + 42530 (R^2 = 0.0102)	-72.4ln(x) + 41814 (R^2 = 4E-05)	$42402e^{-4E-05x}$ (R^2 = 0.0097)
Mn	-0.0495x + 1261 (R^2 = 0.0028)	-55.19ln(x) + 1587 (R^2 = 0.005)	$1144.3e^{2E-05x}$ (R^2 = 0.0028)
Ni	-0.0305x + 1343 (R^2 = 0.0009)	48.959ln(x) + 985 (R^2 = 0.003)	$1412.6e^{-2E-04x}$ (R^2 = 0.0003)
Pb	0.0003x + 14.12 (R^2 = 0.002)	-0.401ln(x) + 17.12 (R^2 = 0.005)	$13.855e^{3E-05x}$ (R^2 = 0.0024)
Zn	-0.0027x + 47.081 (R^2 = 0.005)	-6.448ln(x) + 88.07 (R^2 = 0.041)	$41.729e^{2E-05x}$ (R^2 = 0.0045)
Steel factory	Linear equation	Logarithmic equation	Exponential equation
Cd	-4E-06x + 0.181 (R^2 = 0.0005)	0.021ln(x) + 0.03 (R^2 = 0.0184)	-
Co	0.0005x + 25.8 (R^2 = 0.0016)	2.33ln(x) + 10.66 (R^2 = 0.0437)	$23.068e^{6E-05x}$ (R^2 = 0.0009)
Cr	-0.0281x + 391(R^2 = 0.0116)	15.76ln(x) + 252.8 (R^2 = 0.005)	$303.09e^{1E-06x}$ (R^2 = 0.0107)
Cu	0.0044x + 16.499 (R^2 = 0.285)	3.412ln(x) - 1.648 (R^2 = 0.233)	$16.661e^{0.0002x}$ (R^2 = 0.2703)
Fe	4.5382x + 27294 (R^2 = 0.2318)	4564ln(x) + 1516 (R^2 = 0.3193)	$26638e^{0.0002x}$ (R^2 = 0.2086)
Mn	0.0364x + 606.8 (R^2 = 0.0606)	37.1ln(x) + 396.9 (R^2 = 0.0857)	$599.07e^{6E-05x}$ (R^2 = 0.0574)
Ni	-0.001x + 258.77 (R^2 = 2E-05)	29.8ln(x) + 56.08 (R^2 = 0.0275)	$181.61e^{0.0001x}$ (R^2 = 0.0005)
Pb	-0.0033x + 7.16 (R^2 = 0.1785)	-4.37ln(x) + 33 (R^2 = 0.4158)	-
Zn	-0.0186x + 107 (R^2 = 0.1492)	-24.68ln(x) + 253 (R^2 = 0.3582)	$101.91e^{-2E-04x}$ (R^2 = 0.1726)

Table S6. Pearson correlation coefficient values for the total element concentrations in the three studied areas.

**Eretria**	**Dist**	**Cd**	**Co**	**Cr**	**Cu**	**Fe**	**Mn**	**Ni**	**Pb**	**Zn**
Distance	1									
Cd	-0.141	1								
Co	-0.783**	0.205	1							
Cr	-0.626**	0.187	0.804**	1						
Cu	0.242	-0.085	-0.284	-0.454	1					
Fe	-0.733**	0.491*	0.881**	0.712**	-0.094	1				
Mn	-0.508*	-0.273	0.788**	0.550*	-0.008	0.583*	1			
Ni	-0.876**	0.302	0.886**	0.693**	-0.420	0.817**	0.497	1		
Pb	-0.262	-0.176	0.483*	0.561*	0.171	0.348	0.595	0.255	1	
Zn	-0.352	0.345	0.306	0.012	0.458	0.471*	0.246	0.273	0.339	1
Domokos	Distance	Cd	Co	Cr	Cu	Fe	Mn	Ni	Pb	Zn
Distance	1									
Cd	-0.240	1								
Co	-0.303	0.131	1							
Cr	-0.466*	-0.120	-0.017	1						
Cu	-0.011	0.140	-0.115	-0.358	1					
Fe	-0.101	0.073	0.573**	0.056	0.220	1				
Mn	-0.053	0.087	0.796**	-0.426	-0.099	0.154	1			
Ni	-0.029	0.068	0.367	0.091	-0.051	0.635**	0.040	1		
Pb	0.047	0.179	-0.090	-0.371	-0.265	-0.579	0.274	-0.253	1	
Zn	-0.072	0.748**	0.250	-0.269	0.258	0.276	0.198	0.196	0.210	1
Steel factory	Distance	Cd	Co	Cr	Cu	Fe	Mn	Ni	Pb	Zn
Distance	1									
Cd	-0.023	1								
Co	0.039	-0.255	1							
Cr	-0.103	-0.280	0.907**	1						
Cu	0.534**	-0.022	0.036	-0.172	1					
Fe	0.481**	-0.187	0.683**	0.556**	0.606**	1				
Mn	0.246	0.076	0.242	0.103	0.698**	0.699**	1			
Ni	-0.005	-0.223	0.942**	0.958**	-0.121	0.594**	0.135	1		
Pb	-0.422*	0.233	-0.423*	-0.365*	-0.312	-0.585**	-0.280	-0.367*	1	
Zn	-0.386*	0.333	-0.413*	-0.365*	-0.113	-0.438*	0.045	-0.385*	0.895**	1

* Significant at the level of $p<0.05$, ** Significant at the level of $p < 0.01$.

Table S7. Descriptive statistics of CF values in Eretria, Domokos and steel factory (industrial area of Volos).

**Eretria**	**Cd**	**Co**	**Cr**	**Cu**	**Fe**	**Mn**	**Ni**	**Pb**	**Zn**
Min	0.66	1.79	3.36	0.09	–	1.28	2.65	0.43	0.51
10th perc	0.66	2.26	4.11	0.14	–	1.54	8.40	0.43	0.54
50th perc	1.00	6.63	11.55	0.25	–	2.02	49.25	0.56	0.75
Average	1.23	6.05	11.85	0.30	–	2.09	42.33	0.56	0.77
90th perc	1.76	10.19	18.79	0.49	–	2.89	72.08	0.71	0.99
Max	1.99	10.94	21.38	0.75	–	3.49	77.13	0.85	1.56
Skewness	0.40	0.00	0.00	1.29	–	1.12	-0.25	0.64	1.93
Domokos	Cd	Co	Cr	Cu	Fe	Mn	Ni	Pb	Zn
Min	1.00	3.24	6.09	0.11	–	1.27	11.40	0.43	0.31
10th perc	1.00	4.63	9.93	0.17	–	1.82	31.05	0.43	0.48
50th perc	1.00	7.33	11.98	0.21	–	2.29	46.19	0.51	0.61
Average	1.09	7.44	13.07	0.22	–	2.53	45.35	0.53	0.64
90th perc	1.36	9.68	15.55	0.29	–	3.47	61.66	0.61	0.75
Max	1.66	12.15	26.22	0.35	–	4.08	62.47	0.77	1.30
Skewness	2.08	0.05	1.53	0.39	–	0.62	-1.06	0.71	1.90
Steel factory	Cd	Co	Cr	Cu	Fe	Mn	Ni	Pb	Zn
Min	0.00	1.06	1.14	0.31	–	0.90	1.84	0.00	0.69
10th perc	0.00	1.36	2.38	0.37	–	1.05	2.78	0.00	0.72
50th perc	0.40	2.26	5.89	0.51	–	1.32	8.90	0.00	1.18
Average	0.43	2.34	6.04	0.55	–	1.35	8.88	0.13	1.23
90th perc	0.78	3.41	11.07	0.77	–	1.68	16.12	0.38	1.67
Max	1.27	4.22	11.98	0.91	–	1.73	18.91	0.93	3.07
Skewness	0.71	0.45	0.35	0.54	–	0.05	0.43	2.21	1.84

Table S8. Descriptive statistics for Pollution Load Index (PLI) for the studied areas.

	**Eretria**	**Domokos**	**Steel factory**
Min	1.14	1.61	0.80
10th perc	1.28	1.91	0.94
50th perc	2.33	2.34	1.65
Average	2.20	2.28	1.61
90th perc	2.92	2.53	2.15
Max	3.19	2.95	2.67
Skewness	–0.31	–0.29	0.04

Table S9. Pearson correlation of PLI index values with distance from suspected pollution source. .

**PLI**	**Pearson correlation**	**Sig.**
Eretria	–0.779	0.000
Domokos	–0.287	0.220
Steel factory	0.075	0.695

Table S10. Varimax rotated factor loading matrix for Eretria sampling area.

Sampling site: Eretria	Initial Eigenvalues			Extraction Sums of Squared Loadings			Rotation Sums of square loadings
Components	Total	% Variance	Cumulative %	Total	% Variance	Cumulative %	Total
1	5.121	51.209	51.209	5.121	51.209	51.209	5.085
2	1.740	17.401	68.610	1.740	17.401	68.610	1.909
3	1.566	15.663	84.273	1.566	15.663	84.273	1.739
4	.689	6.885	91.158				
5	.389	3.890	95.048				
6	.277	2.774	97.823				
7	.117	1.175	98.998				
8	.064	.642	99.640				
9	.029	.290	99.930				
10	.007	.070	100.000				

Eretria—Components	Component Correlation Matrix		
	PC 1	PC 2	PC 3
PC 1	1.000	0.191	0.112
PC 2	0.191	1.000	0.067
PC 3	0.112	0.067	1.000
Kaiser–Meyer–Olkin Measure of Sampling Adequacy			0.553
Bartlett's Test of Sphericity			*P* < 0.001

Table S11. Rotated component matrix of principal component analysis for heavy metals, component correlation matrix, KMO and Bartlett's test for Eretria sampling sites.

Eretria—Variables	Principal components		
	**PC 1**	**PC 2**	**PC 3**
Distance	−0.858	0.004	0.023
Cd	0.373	0.242	−0.841
Co	0.955	−0.014	0.140
Cr	0.877	−0.276	0.170
Cu	−0.508	0.862	0.173
Fe	0.896	0.224	−0.161
Mn	0.579	0.095	0.621
Ni	0.966	−0.122	−0.136
Pb	0.332	0.300	0.635
Zn	0.215	0.862	−0.148
**Eretria—Components**	**Component Correlation Matrix**		
	**PC 1**	**PC 2**	**PC 3**
PC 1	1.000	0.191	0.112
PC 2	0.191	1.000	0.067
PC 3	0.112	0.067	1.000
Kaiser–Meyer–Olkin Measure of Sampling Adequacy			0.553
Bartlett's Test of Sphericity			$p < 0.001$

Table S12. Varimax rotated factor loading matrix for Domokos sampling sites.

Sampling site: Domokos	Initial Eigenvalues			Extraction Sums of Squared Loadings			Rotation Sums of square loadings
**Components**	**Total**	**% Variance**	**Cumulative %**	**Total**	**% Variance**	**Cumulative %**	**Total**
1	2.703	27.030	27.030	2.703	27.030	27.030	2.284
2	2.174	21.744	48.774	2.174	21.744	48.774	2.140
3	1.615	16.152	64.926	1.615	16.152	64.926	2.158
4	1.479	14.787	79.712	1.479	14.787	79.712	1.721
5	.961	9.607	89.319				
6	.496	4.956	94.276				
7	.255	2.549	96.825				
8	.179	1.793	98.618				
9	.109	1.086	99.704				
10	.030	.296	100.000				

Table S13. Rotated component matrix of principal component analysis for heavy metals, component correlation matrix, KMO and Bartlett's test for Domokos sampling sites.

**Domokos—Variables**	**Principal components**			
	**1**	**2**	**3**	**4**
Distance	–0.001	–0.039	–0.372	0.799
Cd	–0.130	–0.077	0.955	–0.223
Co	0.387	0.855	0.023	–0.138
Cr	0.142	–0.276	–0.128	–0.860
Cu	0.344	–0.395	0.385	0.447
Fe	0.907	0.230	0.065	0.029
Mn	–0.036	0.944	–0.035	0.211
Ni	0.672	0.226	0.015	–0.056
Pb	–0.812	0.298	0.228	0.075
Zn	0.043	0.068	0.889	0.036
Domokos—Components	Component correlation matrix			
	PC 1	PC 2	PC 3	PC 4
PC 1	1.000	0.018	0.112	–0.077
PC 2	0.018	1.000	0.215	–0.038
PC 3	0.112	0.215	1.000	0.180
PC 4	–0.077	–0.038	0.180	1.000
Kaiser–Meyer–Olkin Measure of Sampling Adequacy				0.514
Bartlett's Test of Sphericity				$p < 0.001$

Table S14. Varimax rotated factor loading matrix for steel factor sampling sites.

**Sampling site: Steel factory**	**Initial Eigenvalues**			**Extraction Sums of Squared Loadings**			**Rotation Sums of square loadings**
**Components**	**Total**	**% Variance**	**Cumulative %**	**Total**	**% Variance**	**Cumulative %**	**Total**
**1**	4.371	43.708	43.708	4.371	43.708	43.708	3.647
**2**	2.381	23.806	67.514	2.381	23.806	67.514	2.977
**3**	1.460	14.598	82.112	1.460	14.598	82.112	2.961
**4**	0.834	8.341	90.454				
**5**	0.550	5.496	95.949				
**6**	0.203	2.030	97.979				
**7**	0.073	0.733	98.712				
**8**	0.073	0.728	99.441				
**9**	0.038	0.377	99.817				
**10**	0.018	0.183	100.000				

Table S15. Rotated component matrix of principal component analysis for heavy metals, component correlation matrix, KMO and Bartlett's test for steel factory sampling sites.

**Steel factory—Variables**	**Principal components**		
	**PC 1**	**PC 2**	**PC 3**
Distance	−0.340	0.509	−0.505
Cd	−0.151	0.262	0.522
Co	0.928	0.082	−0.066
Cr	0.977	−0.119	−0.056
Cu	−0.243	0.930	−0.013
Fe	0.481	0.699	−0.127
Mn	0.176	0.956	0.352
Ni	0.965	−0.052	−0.056
Pb	−0.098	−0.175	0.802
Zn	−0.079	0.131	0.968
Steel factory—Components	Component correlation matrix		
	PC 1	PC 2	PC 3
PC 1	1.000	0.169	−0.313
PC 2	0.169	1.000	−0.347
PC 3	−0.313	−0.347	1.000
Kaiser–Meyer–Olkin Measure of Sampling Adequacy			0.653
Bartlett's Test of Sphericity			$p < 0.001$

Table S16. Potentially toxic elements in cereal shoot tissues (mg kg^{-1}) in Eretria, Domokos and steel factory (industrial area of Volos).

**Eretria**	**Cd**	**Co**	**Cr**	**Cu**	**Fe**	**Mn**	**Ni**	**Pb**	**Zn**
Min	BDL[a]	BDL	BDL	BDL	0.00	0.00	BDL	BDL	0.00
10th perc	BDL	BDL	BDL	BDL	0.00	0.00	BDL	BDL	0.00
50th perc	BDL	BDL	BDL	BDL	17.79	5.20	BDL	BDL	10.21
Average	BDL	BDL	BDL	BDL	26.23	7.00	BDL	BDL	10.26
90th perc	BDL	BDL	BDL	BDL	58.07	14.52	BDL	BDL	20.38
Max	BDL	BDL	BDL	BDL	73.27	26.98	BDL	BDL	24.49
Skewness	NA[b]	NA	NA	NA	0.49	1.37	NA	NA	0.07
Domokos	Cd	Co	Cr	Cu	Fe	Mn	Ni	Pb	Zn
Min	BDL	BDL	BDL	BDL	0.00	0.00	BDL	BDL	0.00
10th perc	BDL	BDL	BDL	BDL	0.00	0.00	BDL	BDL	0.00
50th perc	BDL	BDL	BDL	BDL	10.95	5.51	BDL	BDL	11.43
Average	BDL	BDL	BDL	BDL	18.94	7.93	BDL	BDL	12.71
90th perc	BDL	BDL	BDL	BDL	36.79	14.16	BDL	BDL	22.40
Max	BDL	BDL	BDL	BDL	109.75	35.45	BDL	BDL	34.41
Skewness	NA	NA	NA	NA	2.70	1.73	NA	NA	0.59
Steel factory	Cd	Co	Cr	Cu	Fe	Mn	Ni	Pb	Zn
Min	NA	NA	25.60	3.41	NA	10.47	NA	NA	20.60
10th perc	NA	NA	54.16	4.18	NA	21.66	NA	NA	24.13
50th perc	NA	NA	144.78	6.47	NA	35.17	NA	NA	39.86
Average	NA	NA	181.77	8.89	NA	33.84	NA	NA	42.05
90th perc	NA	NA	289.33	17.54	NA	43.58	NA	NA	72.38
Max	NA	NA	794.61	32.65	NA	61.57	NA	NA	76.52
Skewness	NA	NA	2.65	2.30	NA	0.03	NA	NA	0.85

a: BDL: Below detection limit.

b: NA: Not appropriate/not available.

Table S17. Potentially toxic elements in cereal grains (mg kg^{-1}) in Eretria, Domokos and steel factory (industrial area of Volos).

**Eretria**	**Cd**	**Co**	**Cr**	**Cu**	**Fe**	**Mn**	**Ni**	**Pb**	**Zn**
Min	BDL[a]	BDL	BDL	BDL	0.00	1.57	BDL	BDL	19.63
10th perc	BDL	BDL	BDL	BDL	0.00	2.00	BDL	BDL	21.05
50th perc	BDL	BDL	BDL	BDL	0.91	7.02	BDL	BDL	26.51
Average	BDL	BDL	BDL	BDL	3.38	9.29	BDL	BDL	27.53
90th perc	BDL	BDL	BDL	BDL	8.52	18.03	BDL	BDL	34.96
Max	BDL	BDL	BDL	BDL	17.03	19.72	BDL	BDL	37.65
Skewness	NA[b]	NA	NA	NA	2.31	0.50	NA	NA	0.37
Domokos	Cd	Co	Cr	Cu	Fe	Mn	Ni	Pb	Zn
Min	BDL	BDL	BDL	BDL	BDL	1.57	BDL	BDL	16.19
10th perc	BDL	BDL	BDL	BDL	BDL	9.14	BDL	BDL	18.41
50th perc	BDL	BDL	BDL	BDL	BDL	17.91	BDL	BDL	27.12
Average	BDL	BDL	BDL	BDL	BDL	16.77	BDL	BDL	27.31
90th perc	BDL	BDL	BDL	BDL	BDL	22.75	BDL	BDL	34.21
Max	BDL	BDL	BDL	BDL	BDL	25.77	BDL	BDL	40.08
Skewness	NA	NA	NA	NA	NA	-0.95	NA	NA	0.02
Steel factory	Cd	Co	Cr	Cu	Fe	Mn	Ni	Pb	Zn
Min	NA	NA	4.46	4.95	NA	5.02	NA	NA	21.20
10th perc	NA	NA	7.10	6.24	NA	12.07	NA	NA	24.50
50th perc	NA	NA	9.68	8.68	NA	21.73	NA	NA	40.39
Average	NA	NA	9.53	9.03	NA	30.92	NA	NA	45.12
90th perc	NA	NA	11.89	11.49	NA	46.08	NA	NA	64.63
Max	NA	NA	14.34	20.92	NA	221.65	NA	NA	109.21
Skewness	NA	NA	-0.09	2.05	NA	4.48	NA	NA	1.89

a: BDL: Below detection limit.

b: NA: Not appropriate/not available.

Table S18. Descriptive statistics of potentially toxic elements extracted using AB-DTPA (ammonium bicarbonate-diethylene-triamine pentaacetic acid, mg kg^{-1}) and percentage of availability of AB-DTPA-extractable elements relative to the pseudo-total elements.

**Eretria**	**Cd**	**Co**	**Cr**	**Cu**	**Fe**	**Mn**	**Ni**	**Pb**	**Zn**
Min	BDL[b]	BDL	0.00	0.00	30.65	10.94	BDL	BDL	BDL
10th perc	BDL	BDL	0.00	0.00	54.24	24.34	1.29	BDL	0.02
50th perc	BDL	1.39	0.00	0.12	176.57	69.74	22.39	BDL	0.27
Average	BDL	2.03	0.00	0.38	177.78	80.20	27.35	BDL	0.45
90th perc	BDL	3.73	0.00	1.03	335.62	143.51	63.83	BDL	1.05
Max	BDL	8.00	0.00	1.52	468.41	186.63	70.54	BDL	1.73
Skewness	NA[c]	1.33	NA	1.32	0.91	0.56	0.66	NA	1.74
%Availability[a]	NA	2.29	NA	3.01	0.39	7.80	2.35	NA	0.84
Domokos	Cd	Co	Cr	Cu	Fe	Mn	Ni	Pb	Zn
Min	BDL	BDL	BDL	0.83	BDL	BDL	BDL	BDL	BDL
10th perc	BDL	0.13	BDL	1.31	17.15	36.01	7.76	BDL	BDL
50th perc	BDL	0.73	BDL	1.64	159.54	58.37	23.15	BDL	0.13
Average	BDL	0.97	BDL	1.71	155.70	67.72	28.10	BDL	0.42
90th perc	BDL	2.22	BDL	2.25	267.64	112.48	43.70	BDL	0.72
Max	BDL	3.52	BDL	2.69	333.43	125.65	77.27	BDL	4.15
Skewness	NA	1.63	NA	0.39	0.08	0.13	1.00	NA	3.95
%Availability[a]	NA	1.11	NA	20.00	0.37	5.74	2.10	NA	0.76
Steel Factory	Cd	Co	Cr	Cu	Fe	Mn	Ni	Pb	Zn
Min	0.04	0.08	0.03	2.20	4.82	15.78	2.32	1.26	1.48
10th perc	0.07	0.15	0.06	3.79	5.15	18.30	3.66	1.57	2.10
50th perc	0.11	0.26	0.13	5.20	7.65	24.34	7.06	4.36	8.66
Average	0.12	0.27	0.14	5.28	9.42	27.01	6.75	5.30	10.45
90th perc	0.20	0.39	0.23	7.11	11.96	40.71	9.89	10.68	22.83
Max	0.26	0.48	0.33	9.22	49.21	48.12	10.80	16.50	49.49
Skewness	0.98	0.38	0.83	0.41	4.65	0.93	-0.01	1.17	2.26
%Availability[a]	56.73	1.11	0.03	25.18	0.03	4.24	3.22	139.06	10.33

a: Percentage of AB-DTPA-extractable elements over pseudo-total elements.

b: BDL: Below detection limit.

c: NA: Not appropriate/not available.

Table S19. Duncan tests for HQ soil ingestion in the three studied areas: adults (**a**), children (**b**).

**HQ adults**				
**Duncan**[a,b,c]				
	N	Subset		
		1	2	3
Volos	270	.023435		
Eretria	162		.055284	
Domokos	180			.063786
Sig.		1.000	1.000	1.000

Means for groups in homogeneous subsets are displayed.
Based on observed means.
The error term is Mean Square (Error) = .001.
a. Uses Harmonic Mean Sample Size = 194.400.
b. The group sizes are unequal. The harmonic mean of the group sizes is used. Type I error levels are not guaranteed.
c. Alpha = 0.05.

(a)

**HQ children**				
Duncan[a,b,c]				
	N	Subset		
		1	2	3
Volos	270	.218868		
Eretria	162		.516044	
Domokos	180			.595239
Sig.		1.000	1.000	1.000

Means for groups in homogeneous subsets are displayed.
Based on observed means.
The error term is Mean Square (Error) = .118.
a. Uses Harmonic Mean Sample Size = 194.400.
b. The group sizes are unequal. The harmonic mean of the group sizes is used. Type I error levels are not guaranteed.
c. Alpha = 0.05.

(b)

Table S20. Duncan tests for Eretria HQ (soil ingestion) adults (**a**) and children (**b**), Domokos HQ adults (**c**) and children (**d**) and steel factory (Volos) HQ adults (**e**) and children (**f**).

HQ soil ingestion, Eretria—adults

Duncan[a,b]

	N	Subset		
		1	2	3
Zn	18	.0002457		
Cu	18	.0003957		
Cr	18	.0006439		
Cd	18	.0006939		
Pb	18	.0059167		
Mn	18	.0098267		
Fe	18		.0835333	
Ni	18		.0840300	
Co	18			.3122667
Sig.		.660	.979	1.000

Means for groups in homogeneous subsets are displayed.

Based on observed means.

The error term is Mean Square (Error) = .003.

a. Uses Harmonic Mean Sample Size = 18.000.

b. Alpha = 0.05.

(a)

HQ soil ingestion, Eretria—children

Duncan[a,b]

	N	Subset		
		1	2	3
Zn	18	.002293		
Cu	18	.003695		
Cr	18	.006008		
Cd	18	.006470		
Pb	18	.055217		
Mn	18	.091706		
Fe	18		.779611	
Ni	18		.784289	
Co	18			2.915111
Sig.		.660	.978	1.000

Means for groups in homogeneous subsets are displayed.

Based on observed means.

The error term is Mean Square (Error) = .269.

a. Uses Harmonic Mean Sample Size = 18.000.

b. Alpha = 0.05.

(b)

HQ soil ingestion, Domokos—adults

Duncan[a,b]

	N	Subset		
		1	2	3
Zn	20	.0002		
Cu	20	.0003		
Cd	20	.0006		
Cr	20	.0007		
Pb	20	.0056		
Mn	20	.0119		
Fe	20		.0809	
Ni	20		.0900	
Co	20			.3838
Sig.		.420	.460	1.000

Means for groups in homogeneous subsets are displayed.

Based on observed means.

The error term is Mean Square (Error) = .002.

a. Uses Harmonic Mean Sample Size = 20.000.

b. Alpha = 0.05.

HQ soil ingestion, Domokos—children

Duncan[a,b]

	N	Subset		
		1	2	3
Zn	20	.001900		
Cu	20	.002741		
Cd	20	.005741		
Cr	20	.006628		
Pb	20	.052640		
Mn	20	.110950		
Fe	20		.754750	
Ni	20		.840300	
Co	20			3.581500
Sig.		.419	.459	1.000

Means for groups in homogeneous subsets are displayed.

Based on observed means.

The error term is Mean Square (Error) = .133.

a. Uses Harmonic Mean Sample Size = 20.000.

b. Alpha = 0.05.

(c) (d)

HQ soil ingestion, steel factory (Volos)—adults

Duncan[a,b]

	N	Subset 1	2	3	4
Cd	30	.000242			
Cr	30	.000328			
Zn	30	.000393			
Cu	30	.000734			
Pb	30	.001338			
Mn	30	.006337			
Ni	30		.017646		
Fe	30			.063363	
Co	30				.120530
Sig.		.185	1.000	1.000	1.000

Means for groups in homogeneous subsets are displayed.

Based on observed means.

The error term is Mean Square (Error) = .000.

a. Uses Harmonic Mean Sample Size = 30.000.

b. Alpha = 0.05.

(e)

HQ soil ingestion, steel factory (Volos)—children

Duncan[a,b]

	N	Subset 1	2	3	4
Cd	30	.0023			
Cr	30	.0031			
Zn	30	.0037			
Cu	30	.0069			
Pb	30	.0125			
Mn	30	.0591			
Ni	30		.1648		
Fe	30			.5915	
Co	30				1.1261
Sig.		.185	1.000	1.000	1.000

Means for groups in homogeneous subsets are displayed.

Based on observed means.

The error term is Mean Square (Error) = .021.

a. Uses Harmonic Mean Sample Size = 30.000.

b. Alpha = 0.05.

(f)

Table S21. Hazard Index (HI) for soil ingestion in the three studied areas.

.	Eretria		Domokos		Steel factory	
	Adult	Child	Adult	Child	Adult	Child
Min	0.17	1.62	0.27	2.49	0.10	0.97
10th perc	0.21	2.00	0.41	3.78	0.14	1.26
50th perc	0.55	5.15	0.60	5.62	0.21	1.95
Average	0.50	4.64	0.57	5.36	0.21	1.97
90th perc	0.78	7.33	0.69	6.40	0.30	2.77
Max	0.80	7.46	0.84	7.89	0.33	3.09
Skewness	-0.18	-0.18	-0.40	-0.40	0.14	0.14

Table S22. Hazard Index for humans from wheat grain consumption.

Eretria	Eretria	Domokos	Industrial Area
Min	0.43	0.39	0.96
10th perc	0.45	0.72	1.24
50th perc	0.62	1.01	1.86
Average	0.69	0.96	2.01
90th perc	0.98	1.18	2.96
Max	1.13	1.26	4.99
Skewness	0.61	-1.06	1.77

References (Only those references not cited in the main text are cited here)

1. Bonelli, M.G., Manni, A. Principal components analysis and spatial analysis integration for enhanced assessment of pollution emission sources. IOP Conf. Series: *Earth Environ. Sci.* **2019,** 227, 062013.
2. Chen, T., Chang, Q., Liy, J., Clevers, J.G.P.W., Kooistra, L. Identification of soil heavy metal sources and improvement in spatial mapping based on soil spectral information: A case study in northern China. *Sci. Total Environ.* **2016**, 565, 155-164.
3. Allafta, H.; Opp, C. Spatio-temporal variability and pollution sources identification of the surface sediments of Shatt Al-Arab River, Southern Iraq. *Sci. Rep.* **2020**, *10*, 6979.
4. Krami, L.M.; Amiri, F.; Sefianian, A.; Shariff, A.R.M.; Tabatabaie, T.; Pradhan, B. Spatial patterns of heavy metals in soil under different geological structures and land uses for assessing metal enrichments. *Environ. Monit. Assess*. **2013**, *185*, 9871–9888.
5. Elhadi, R.E.; Abdullah, A.M.; Abdullah, A.H.; Hanan Ash'aari, Z.; Kura, N.U.D.Y.G.; Adamu, A. Source identification of heavy metals in particulate patter (PM10) in a Malaysian traffic area using multivariate techniques. *Pol. J. Environ. Stud.* **2017**, *26*, 2523–2532.
6. Wu, J., Teng, Y., Lu, S., Wang, Y., Jiao, X. Evaluation of soil contamination indices in a mining area of Jiangxi, China. *PLoS* **2014**, One, 9, 112917.
7. Olowoyo, O.J., Odiwe, I.J., Mkolo, M.N., Macheka, L. Investigating the concentrations of different elements in soil and plant composition from a mining area. *Polish Journal of Environmental Studies* **2013**, *22*, 1135-1141.
8. Demková, L.; Jezný, T.; Bobuľská, L. Assessment of soil heavy metal pollution in a former mining area—Before and after the end of mining activities. *Soil Water Res.* **2017**, *12*, 229–236.
9. Huang, Y.; Li, T.; Wu, C.; He, Z.; Japenga, J.; Deng, M.; Yang, X. An integrated approach to assess heavy metal source apportionment in peri-urban agricultural soils. *J. Hazard. Mater.* **2015**, *299*, 540–549.
10. Crispo, M.; Dobson, C.M.; Blevins, S.R.; Meredith, W.; Lake, A.J.; Edmondson, L.J. Heavy metals and metalloids concentrations across UK urban horticultural soils and the factors influencing their bioavailability to food crops. *Environ. Pollut*. **2021**, *288*, 117960.
11. Tanzeem-ul-Haq, H.S.; Rasool, B.; Ehtisham-ul-Haque, S.; Saif, S.; Zafar, S.; Younis, T.; Akhtar, I.; Jafri, L.; Iqbal, N.; Masood, N.; et al. Chitosan with bentonite and biochar in Ni-affected soil reduces grain Ni concentrations, improves soil enzymes and grain quality in lentil. *Minerals* **2021**, *11*, 11.
